# Spatio-Temporal Neural Changes After Task-Switching Training in Old Age

**DOI:** 10.3389/fnagi.2019.00267

**Published:** 2019-10-15

**Authors:** Sandra Dörrenbächer, Chiara Schütz, Marc Woirgardt, C. Carolyn Wu, Hubert D. Zimmer, Jutta Kray

**Affiliations:** ^1^Department of Psychology, Development of Language, Learning and Action, Saarland University, Saarbrücken, Germany; ^2^Department of Biological and Clinical Psychology, University of Trier, Trier, Germany

**Keywords:** aging, cognitive control impairments, task-switching training, fMRI, context updating, delayed recognition, spatio-temporal overlap

## Abstract

In the present study, we aimed at examining selective neural changes after task-switching training in old age by not only considering the spatial location but also the timescale of brain activation changes (i.e., sustained/block-related or transient/trial-related timescales). We assigned a sample of 50 older adults to a task-switching training or an active single-task control group. We administered two task paradigms, either sensitive to transient (i.e., a context-updating task) or sustained (i.e., a delayed-recognition working-memory task) dynamics of cognitive control. These dynamics were captured by utilizing an appropriate event-related or block-related functional magnetic resonance imaging design. We captured selective changes in task activation during the untrained tasks after task-switching training compared to an active control group. Results revealed changes at the neural level that were not evident from only behavioral data. Importantly, neural changes in the transient-sensitive context updating task were found on the same timescale but in a different region (i.e., in the left inferior parietal lobule) than in the task-switching training task (i.e., ventrolateral PFC, inferior frontal junction, superior parietal lobule), only pointing to temporal overlap, while neural changes in the sustained-sensitive delayed-recognition task overlapped in both timescale and region with the task-switching training task (i.e., in the basal ganglia), pointing to spatio-temporal overlap. These results suggest that neural changes after task-switching training seem to be critically supported by the *temporal* organization of neural processing.

## Research Highlights

- Examination of selective neural changes after task-switching training in old age by considering both the spatial location and the timescale (i.e., sustained/block-related or transient/trial-related timescales) of brain-activation changes.
- As compared to the training task, examination of whether the amount of brain changes in untrained tasks after task-switching training depends not only on spatial (i.e., same brain region) but also on temporal overlap (i.e., same timescale).
- Selective neural changes after task-switching training in an untrained transient-sensitive context updating task were found in the inferior parietal lobule (i.e., on the same timescale but in a different region than in the task-switching training task), while neural changes in an untrained sustained-sensitive delayed-recognition task were found in the basal ganglia (i.e., on overlapping region and timescale with the task-switching training task).
- Discussion of the critical role of the *temporal* organization of neural processing for the investigation of training-induced brain-activation changes.

## Introduction

One core component of executive control is the ability to schedule and sustain multiple goals at the same time or in rapid alternation. It is now well established that this ability is trainable by specific interventions, such as task switching or multi-tasking training, which may be particularly relevant for older adults that show genuine impairments in executive behavior (Karbach and Kray, 2009; Anguera et al., 2013; Karbach and Verhaeghen, 2014). Recent neuroimaging studies suggest fundamental changes at the neural level underlying this age-related decline in executive control. Specifically, research revealed age-associated brain activation changes on transient (i.e., brief, trial-related) and sustained (i.e., enduring, block-related) timescales in distributed cortical and sub-cortical networks (Dennis et al., 2007; Jimura and Braver, 2009), such as in the prefrontal cortex (e.g., frontal pole: Brodmann area (BA) 10; dorsolateral prefrontal cortex (PFC): BA 9, 46; ventrolateral PFC: BA 44, 45; inferior frontal junction: intersection of BA 6,8,9, and 44; anterior cingulate: BA 6, 8, and 32) as well as in parietal circuits (inferior parietal lobule: BA 39,40; superior parietal lobule: BA 5, 7) and in subcortical areas in the basal ganglia (BG), including the putamen and the caudate head (see Dosenbach et al., 2006; Dosenbach et al., 2007, 2008; Gold et al., 2010; Gazes et al., 2012; Kim et al., 2012; Nee et al., 2013). Hence, interventions aiming at improving task switching behavior in older adults, such as the present one, should be evaluated by their impact on the neural dynamics underlying performance changes.

In our recent study (Dörrenbächer et al., 2017a,b), we already examined such neural plasticity in brain activity dynamics after task-switching training in older adults during the task-switching task. Specifically, we applied a hybrid event-related-/block fMRI design (Visscher et al., 2003) to investigate not only spatial but also temporal dynamics of neural mechanisms underlying changes after training in task switching compared to a single-task active control regime in older adults. After task-switching training, we found selective changes in the task-switching training task (a) for trial-related brain activation in fronto-lateral (i.e., bilateral ventrolateral PFC and inferior frontal junction) and parietal regions (i.e., left superior parietal lobule), but (b) for block-related brain activation in the basal ganglia bilaterally (see section “Materials and Methods” for details). Hence, our results revealed *spatio-temporal interactions* in training-induced neural changes. These spatially dissociable changes of trial- versus block-related brain activation were both related to improvement of task switching behavior. However, considering the behavioral level alone, training group differences were altogether small. Hence, a second important insight of that study was that neural mechanisms might reveal more subtle training-induced effects that we may not become aware of from only examining the behavioral data.

Based on these findings, the present study aimed to explore further the nature and scope of such neural plasticity beyond behavioral changes in untrained task paradigms tapping into the updating of task sets and the inhibition of irrelevant task contents.

Empirical evidence for transfer of functional brain changes after executive-control training in older adults is scarce so far. Heinzel et al. (2016) found a change in the spatial distribution of brain activation across canonical control networks in older adults. In that study, older adults practiced working-memory updating on an adaptive n-back training task. The authors revealed a selective decrease of neural activation in the right caudal superior frontal sulcus for both the trained n-back task as well as for an untrained Sternberg task for the older adult training group as compared to a no-contact control group.

However, one caveat of that study is that the researchers only included *passive controls*, so that unspecific effects associated with the training regime might have influenced changes in the treatment group. Therefore, in the present study, we included a control group that performed the same task as the treatment group but practiced the task in single-task conditions putting lower demands on executive control than mixed-task conditions (see also Kray and Dörrenbächer, 2019).

Moreover, Heinzel et al. (2016) investigated neural transfer in older adults only as a function of *spatial overlap* across cognitive-control networks. Indeed, neural changes in untrained tasks are usually assumed to occur if activation changes associated with the trained and untrained tasks rely on the same cognitive processes and on spatially overlapping brain regions (Dahlin et al., 2008). However, it has not yet been discussed whether the amount of neural changes in untrained tasks may also depend on overlapping transient or sustained timescales of the involved neural processes, hence on *temporal overlap*.

To summarize, the aims of the present study were twofold: first, to investigate whether task-switching training as compared to active-control single-task training may induce neural changes above and beyond behavioral changes in untrained cognitive tasks by considering both the spatial location and the temporal dynamics of brain-activation changes; and second, to investigate the spatial and temporal overlap of these changes with changes in the training task (Dörrenbächer et al., 2017a,b).

We administered two task paradigms different from the training task: one was sensitive to transient dynamics (i.e., a context-updating task, adapted from Schmitt et al., 2014) and the other was sensitive to sustained dynamics of task switching behavior (a delayed-recognition working-memory task, adapted from Clapp et al., 2010). The context-updating task requires rapidly switching and updating the current task from trial to trial (see also Lenartowicz et al., 2010). Hence, this task taps into local, transient processing requirements on executive control (cf. Marí-Beffa and Kirkham, 2014) and, therefore, was modeled with an event-related fMRI design, capturing transient brain activation dynamics. The latter delayed-recognition working-memory task requires the ability to sustain multiple task-set representations throughout a certain delay period (i.e., sustained task-set maintenance) and to select between them (i.e., sustained scheduling at the task-set level; see section “Materials and Methods” for details). Hence, this task may tap into global, sustained processing requirements on executive control (cf. Marí-Beffa and Kirkham, 2014), thus was implemented into a block-related fMRI design capturing sustained brain activation dynamics.

Regarding our first study goal, we were specifically interested in selective neural changes in the magnitude of transient activation during the context-updating task or of sustained activation during the delayed-recognition task after task-switching training compared to single-task control training. The neural signature of optimal task processing is still a matter of debate. One candidate approach is that of neural efficiency (e.g., Neubauer and Fink, 2009), proposing reductions in the magnitude of brain activation (i.e., task energy) consumed in performing a given task associated with performance improvement. In contrast, the cortical-effort approach suggests increased brain activation as a signature of an increased capability to recruit task-relevant neural resources (for a review, see Barulli and Stern, 2013). Given the dissent in predictions of directional changes of brain activation associated with behavioral improvement, the present study focused on task-beneficial changes in the *magnitude* of brain activation after training, while the direction of change remained an open question.

Regarding our second study goal, to systematically investigate spatial and temporal overlap of changes in activation magnitude during the trained and untrained tasks (cf. Dörrenbächer et al., 2017a,b), we tested one of three likely outcomes based on a region-of-interest (ROI) analysis approach (these potential outcome patterns should be considered as exploratory).

One likely outcome would suggest only *spatial overlap (i.e., same ROIs but different timescale)*, hence an overlap in ROIs that had proven sensitive to the other timescale in the task-switching training task. Given this overlap criterion, the transient-sensitive context-updating task would show changes in those ROIs that had been sensitive to changes in sustained activation in the task-switching training task (i.e., in basal ganglia; and vice versa for the sustained-sensitive delayed-recognition task: in ventrolateral PFC, inferior frontal junction, superior parietal lobule, cf. Dörrenbächer et al., 2017a,b).

Another likely outcome would suggest only *temporal overlap (i.e., same timescale but different ROIs)*, hence an overlap in changes on sustained or transient activation in canonical control regions outside those obtained for the task-switching training task (e.g., frontal pole, dorsolateral PFC, anterior cingulate, inferior parietal lobule, cf. Dörrenbächer et al., 2017a,b).

A third likely outcome would suggest *spatio-temporal overlap (i.e., same ROI and same timescale)*, hence an exact reproduction of patterns found for the task-switching training task. Given this overlap criterion, the transient-sensitive context-updating task would show changes in exactly those regions that had also been sensitive to changes in transient activation in the task-switching training task (i.e., in ventrolateral PFC, inferior frontal junction, superior parietal lobule; and vice versa for the sustained-sensitive delayed-recognition task: in basal ganglia, cf. Dörrenbächer et al., 2017a,b).

## Materials and Methods

### Participants

The required sample size for the overall training study (cf. Dörrenbächer et al., 2017a,b) was calculated by means of an *a priori* power analysis using G^∗^Power based on variance-analytical effect sizes and correlation estimates (Faul et al., 2009). Our sample size considerations were twofold: First, we wanted to have a sufficiently large sample to detect group-differential changes in brain activation. Second, we wanted to have a sufficiently large sample to detect a possible correlation between brain changes and behavioral improvement. We derived *a priori* effect-size estimates from one of the few existing neuroimaging studies on cognitive control training in older adults that applied a similar training design (albeit with dual-task training, Erickson et al., 2007). Based on the *F*-values obtained in this study for the group by session interaction from ROI analyses on canonical PFC regions, we re-calculated partial eta-squared effect size estimates incorporating the correlation between paired measures (Prajapati et al., 2010; Lakens, 2013), ranging between η*^2^*_*partial*_ = 0.28 – 0.31. Brain-behavior correlations between ROI activation changes and behavioral performance changes from pretest to posttest ranged in this study from *r* = 0.62 to 0.69. The necessary total sample size to detect the smallest variance-analytical effect of η^2^_*partial*_ = 0.28 was estimated to be 12. However, the sample size to detect the smallest correlation of *r* = 0.62 (one-tailed) was estimated to be 23 for each separate training group. Due to some unexpected drop-out during transfer measurement (see below), the effective sample sizes for the analysis of the untrained cognitive tasks in the present study ranged between 20 and 24 participants per group.

All participants were recruited by announcements in a local newspaper and from a subject pool of Saarland University. The initial sample consisted of 60 older adults and 30 younger adults, whereby data of the latter younger-adult group are not part of the present study and thus will not be described here in more detail. Older adults were equally assigned to either a high-demanding task-switching training group (TG) or a low-demanding single-task active control group (CG) based on a careful *a priori* matching procedure (see below). Seven older adults in the CG and nine older adults in the TG had to be excluded from the analyses due to the following reasons: they were not willing to undergo the fMRI scanning session after the mock-like simulation on the familiarization session (*n* = 6); incomplete recording sessions (*n* = 3); structural anomalies detected from the T1-weighted scan (i.e., benign cysts that had, however, caused profound signal loss within critical ROI areas, *n* = 2); outlying task switching behavior more than 3 SD beyond average performance of all older adults during at least one recording session (*n* = 3); or technical failures during the fMRI scanning procedure or accidental data loss (*n* = 2). In order to avoid skewing of participant numbers per group, six extra older participants were recruited in a second enrollment to align sample sizes. The final effective sample consisted of 50 older adults, with their age ranging from 61 to 79 years (TG: *n* = 25; *mean age* = 67.80 years, *SD* = 3.85 years, 11 women; CG: *n* = 25; *mean age* = 69.92 years, *SD* = 4.53 years, 14 women). For the analysis of the untrained tasks, some additional participants had to be excluded due to outlying performance or accidental data loss in the specific task paradigm, resulting in an effective sample size of *n* = 20 (CG) and *n* = 24 (TG) participants (context-updating task) and *n* = 21 participants per group (delayed-recognition task).

The two training groups were matched prior to the intervention according to a set of criteria (i.e., age, gender, perceptual speed as measured by the Digit-Symbol Substitution Test (DSST, Wechsler, 1955), verbal knowledge as measured by the Spot-a-Word-Test (SWT, Lehrl, 1977), fluid reasoning as measured by Raven’s progressive matrices (Raven, 1998), baseline speed and accuracy as well as performance costs in the task-switching training task; all *p’s* > 0.14). To obtain a ‘global fit index of matching goodness,’ we calculated iteratively (i.e., for each newly recruited participant) the grand average of these variables that should be as similar as possible, while the sum of SDs of the given variables should be lowest possible, across both groups.

All subjects were native German speakers, right-handed as assessed by the Edinburgh Inventory (Oldfield, 1971), reported normal or corrected-to-normal vision and hearing, no history of any neurological or psychiatric diseases, and reported good physical and mental health. In an adapted version of the DemTect screening (Kessler et al., 2000), all participants scored ten points or higher (scores < nine points indicate mild symptoms of cognitive impairment, and scores ≤ five points indicate suspected clinically relevant symptoms of dementia). The two training groups did not differ in their DemTect scores (*p* = 0.93) nor in any other covariate (all *p’s* > 0.32). For an overview about demographics as a function of training group, see Table 1. Prior to the investigation, all participants were informed about the study procedure and provided written informed consent, in accordance with the protocols approved by the local ethics committee. They were reimbursed for participating in the study with eight Euros per hour, and for travel expenses with a flat fee of 20 Euros.

**TABLE 1:** Means and standard deviations for demographic information and neuropsychological control tests measured at the familiarization and pretest sessions as a function of training group (single-task active control group/CG, task-switching training group/TG).

	**Group**			
	**Single-task active control group (CG)**		**Task-switching training group (TG)**	
	***M***	***SD***	***M***	***SD***
Age (years)	69.92	4.53	67.80	3.85
Education (years)	13.20	5.94	14.00	5.17
Physical health (self-rating)	2.20	0.71	2.16	0.69
Mental health (self-rating)	2.08	0.57	1.76	0.60
Perceptual fluency (DSST)	44.24	9.32	47.04	9.86
Verbal knowledge (SWT)	26.88	3.81	27.56	2.57
Fluid reasoning (Raven’s progressive matrices)	10.08	4.66	10.84	5.76
Dementia Screening (DemTect)	14.16	1.55	14.12	1.64

*Unless reported otherwise, mean values indicate number of correct items. Data from the complete sample (*n* = 50) are reported. The effective sample sizes diverged for the different untrained tasks (context-updating task: *n*(CG) = 20; *n*(TG) = 24; delayed-recognition task: *n*(CG) = 21; *n*(TG) = 21). DSST = Digit Symbol Substitution Test; SWT = Spot-a-Word Test; M = mean; SD = standard deviation.*

### Procedure

In order to evaluate the impact of varied training demands on the amount of behavioral and neural changes, the present study applied a pretest-training-posttest design. Both groups received one familiarization session and two pretest and posttest sessions. Pretest and posttest sessions had similar structure and content, including baseline measurement of task switching performance (that is, performance in the training task) and of the performance on a battery of two untrained cognitive tasks that are of critical importance for the present study and will be detailed further below. At pre- and posttest, we incorporated firstly electroencephalographic (EEG; approximately 180 min) and secondly fMRI measurements (approximately 150 min) to obtain neural indicators of cognitive-control performance. In the present study, only fMRI results will be reported, and therefore the EEG procedures and analyses will not be described in detail. Between pretest and posttest sessions, the older adults underwent eight training sessions spaced over 4 weeks. The training comprised eight sessions (each taking approximately 60 min) of practice in either low-demanding single-task blocks (CG) or in high-demanding mixed-task blocks (TG). Participants were tested individually, either by one (at training) or two experimenters (at pre- and posttest measurements).

### Familiarization Session

In the familiarization session, participants filled out the informed consent and a short demographic questionnaire and performed cognitive tests from the fluid and crystallized domain of intelligence. They were familiarized with the fMRI procedure in a mock scanner (i.e., an environment with the approximate appearance and acoustics of the fMRI procedure) and thereafter decided if they wanted to continue participating in the study.

### Training Procedure (Behavioral)

**FIGURE 1:**
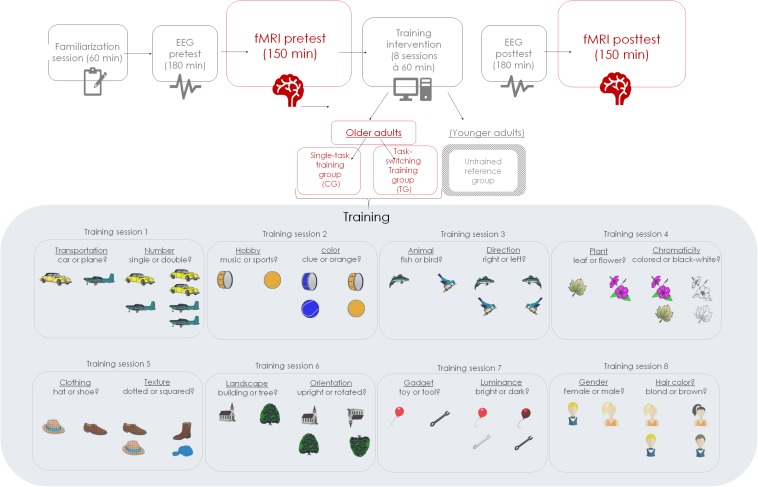
Schematic of the study design of the overall study project.

The task switching paradigm applied during behavioral training was similar to the one of Karbach and Kray (2009), albeit a cued paradigm variant. Different stimulus material and different tasks were used in each session given that training variability may enhance the scope of training transfer (Karbach and Kray, 2009). The order of the different training-task sets across sessions was kept constant for all participants. Participants were asked to categorize pictures^1^ by means of a left or right response button according to a semantic task (e.g., fruit or vegetable?), or a perceptual task (e.g., small or large size?). Critically, response formats were overlapping for both task sets to increase the demand on executive control (cf. Kray and Fehér, 2017). The semantic and perceptual task sets were (see also Figure 1): in the first session, transportation (car or plane?) and number task (single or double?); in the second session, hobby (music or sports?) and color task (blue or orange?); in the third session, animal (fish or bird) and direction task (right or left?) in the fourth session, plant (leaf or flower?) and chromaticity task (colored or black and white?); in the fifth session, clothing (hat or shoe?) and texture task (dotted or squared?); in the sixth session, landscape (building or tree?) and orientation task (upright or rotated?); in the seventh session, gadget (toy or tool?) and luminance task (bright or dark?); and in the eight session, gender (female or male?) and hair-color task (blond or brown?). In the CG condition, participants were required to practice only low-demanding single-task blocks, while in the TG condition, participants practiced high-demanding mixed-task blocks. Each training session amounted to 60 min each and took place twice a week.

In each training session, participants were asked to work first through two practice blocks and then to perform ten experimental blocks, containing 41 trials, with one start trial, 20 repeat trials (i.e., repetition of task-set within the mixed block) and 20 switch trials (i.e., change of task-set within the mixed block) in random order. Each block was equal regarding stimulus and response type, and each mixed-task block (TG) was equal regarding task type. Stimulus-response assignments were counterbalanced across participants, and so was the order of single-task blocks (CG).

Within each trial, the sequence of events was identical for single- and mixed-task trials. Each trial started with a fixation cross in the center of a white screen, lasting 200 ms, followed by a task cue lasting 800 ms. The task cue indicated the next task by the first two letters of the task labels. The cue was followed by an inter-stimulus interval (ISI) of 800 ms. Next, the probe picture appeared, for which responses were recorded. Probe duration was self-paced with a maximum presentation time of 1,800 ms (1 TR) and an extended response deadline of 3,600 ms after probe onset. At the end of each block, participants received feedback about their mean reaction time (RT) and the achieved number of correct responses. Participants were asked to respond as quickly and as accurately as possible with their index fingers.

### Pretest and Posttest Procedure (Including fMRI Measurement)

At pre- and posttest, we firstly measured the performance in the task-switching training task: we applied an adapted, cued version of the paradigm of Karbach and Kray (2009), which was implemented into a mixed epoch-/event-related fMRI design (see also Braver et al., 2003; Jimura and Braver, 2009) allowing for the dissociation of both transient and sustained brain dynamics within the same paradigm (Visscher et al., 2003; Petersen and Dubis, 2012). A detailed description of this specific paradigm variant can be found in Dörrenbächer et al. (2017a,b). However, given that the primary interest of the present study was on the dynamics of neural changes in untrained cognitive tasks, we will focus here on the respective untrained fMRI tasks and describe them in greater detail.

**FIGURE 2:**
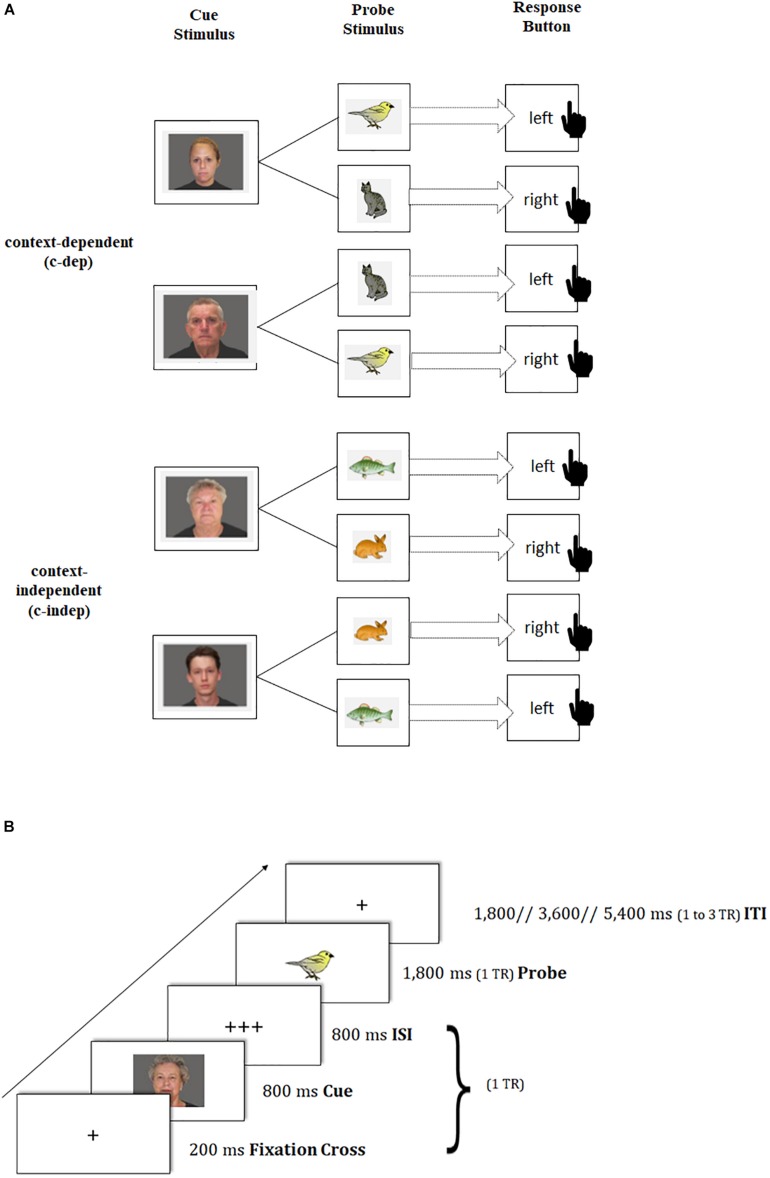
Schematic of the transient-sensitive context-updating paradigm. **(A)** Assignment of cue and probe pictures to response keys on context-independent (c-indep) and context-dependent (c-dep) trials. The black-hand symbol signals the requirement of a manual response. **(B)** Trial procedure and presentation times of stimuli. TR = repetition time; ISI = inter-stimulus interval; ITI = inter-trial interval.

To capture *transient* dynamics underlying neural changes of task-switching training, we provided participants with a modified version of the AX continuous performance task (Braver et al., 2005; Lenartowicz et al., 2010), which was adapted from the study by Schmitt et al. (2014) using pictures instead of letters as stimuli (see also Figure 2A). In this paradigm, participants were presented in each trial with a face picture (cue stimulus: a young or old face, which was either male or female^2^) that was followed by an animal picture (probe stimulus: bird, cat, fish, or rabbit^3^), where the latter had to be responded to by a left or right button press. The cue-probe combinations were presented in two types of conditions: context-dependent (c-dep) and context-independent (c-indep) trials. On *c-dep* trial conditions, the correct answer to the probe stimulus was dependent on the preceding cue information. For instance, participants had to press the left button in response to the bird stimulus, and the right button in response to the cat stimulus, if the preceding cue picture had been a young woman. If the same probes, however, followed the cue picture of an old man, the response mappings had to be reversed. In contrast, on *c-indep* trial conditions, correct responses to the probe stimuli were independent of the preceding cue. Hence, subjects always had to press the same response button to a given probe stimulus (e.g., left button press for the fish picture), irrespective of the preceding cue (i.e., old-woman or young-man picture). Importantly, c-dep as compared to c-indep conditions (further referred to as ‘*context-updating costs*’) put high demands on the flexible updating of stimulus-response mappings from trial to trial, thus pointing to transient processing dynamics.

Participants completed three practice blocks of 16 trials each. The first practice block contained only c-indep trials, followed by a practice block of only c-dep trials, followed by an intermixed practice block. Afterward, participants performed two intermixed experimental blocks, each containing 41 trials, with one start trial, 20 c-indep, and 20 c-dep trials in random order. Each block was equal regarding stimulus and response type. Stimulus-response assignments were counterbalanced across participants. The same tasks were used at pre- and posttest, but with different stimulus sets. Each task block was framed by two fixation blocks of 32.4 s each. The total duration of the task amounted to approximately 15 min (excluding practice blocks, instruction periods, and breaks), and was split into two scanning runs.

Trial events were synced to the repetition time (TR) of two successive scanner pulses, hence locked to multiples of TR = 1,800 ms in the present study (see also Figure 2B). Each trial started with the presentation of a fixation cross for 200 ms, followed by the cue stimulus (800 ms) and a blank interstimulus interval (800 ms), adding up to 1 TR. Subsequently, the probe stimulus was presented for 1,800 ms. Participant’s responses were recorded up to a maximum of 3,600 ms after probe onset. If participants responded faster than 1,800 ms, a blank was inserted that filled up the time until the next scanner pulse was applied (i.e., 1,800 ms minus individual response time), so that subsequent event onsets were realigned with the trigger pulse. The inter-trial interval (ITI) was presented variably, either for 1,800 ms (1 TR), 3,600 ms (2 TR) or 5,400 ms (3 TR). Our ITI durations followed a geometric distribution (Ashby, 2011). At the end of each block, participants received feedback about their mean reaction time (RT) and the achieved number of correct responses. Participants were asked to respond as quickly and as accurately as possible with their index fingers.

**FIGURE 3:**
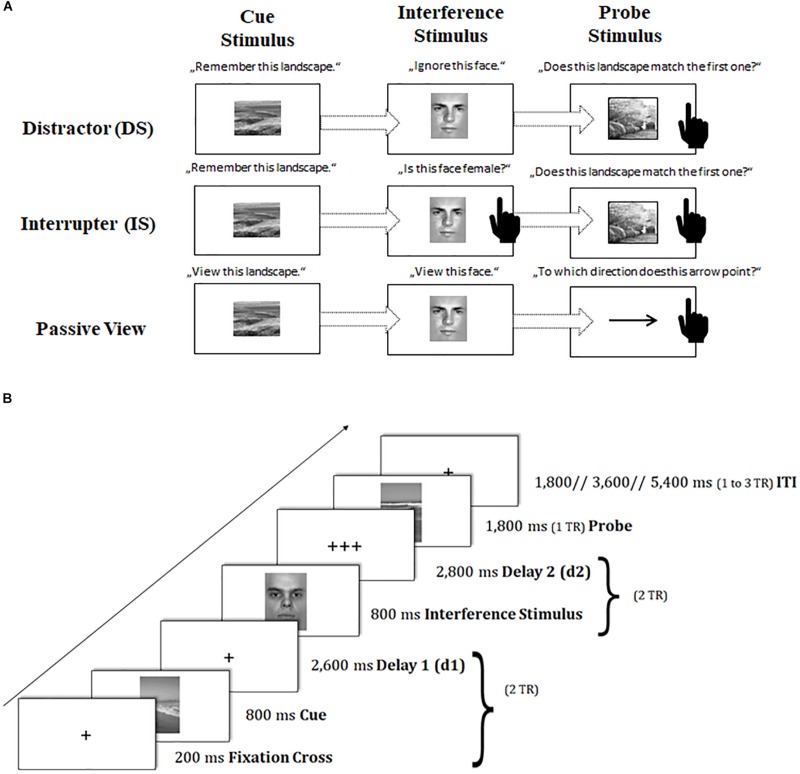
Schematic of the sustained-sensitive delayed-recognition paradigm. **(A)** Sequence of events and task instructions in the cue, interference period for the distractor (DS), interrupter (IS), and passive-view (PV) condition. The black-hand symbol signals the requirement of a manual response. **(B)** Trial procedure and presentation times of stimuli. TR = repetition time. d = delay interval; ITI = inter-trial interval.

To capture *sustained* dynamics underlying neural changes of task-switching training, we provided participants with a modified version of the delayed-recognition task paradigm of the Gazzaley Lab (Gazzaley et al., 2007, 2008; Clapp et al., 2010, 2011). In this paradigm, participants had to memorize a picture of a landscape^4^ (cue landscape) and to maintain it throughout a certain delay period, until a second landscape picture (probe landscape) appeared requiring a recognition match/non-match decision based on a left or right button press. Critically, during the delay period, an interfering face stimulus^5^ was interspersed that required different kinds of operations (see also Figure 3A):

In the *distraction* condition (DS), participants were asked to treat the interfering face stimulus as completely irrelevant information and to suppress or ignore it.

In contrast, in the *interruption* condition (IS), participants were asked to perform a secondary judgment task, where they also had to classify the gender of the interfering face: in a *catch IS trial* (10% of IS trials), where a female picture was presented^6^, participants had to respond with an additional button press, while in a *non-catch IS* trial (the remaining proportion of IS trials), participants should not respond. The catch IS trials were removed from further analyses because they were confounded by a motor response as compared to the other interfering IS stimuli. The IS condition as compared to the DS condition puts additional demands on the scheduling of multiple task sets.

A *passive viewing* condition (PV) served as a baseline condition, where participants were asked to only passively view both the cue landscape and the interfering face stimulus. The probe landscape was replaced here by a probe arrow pointing left or right. Participants had to simply classify the arrow’s direction in this task condition. The PV condition was designed to control non-memory engagement in this task, requiring a simple perceptual response instead of a delayed-recognition decision.

Importantly, all conditions were presented in a block-wise fashion: DS and IS blocks as compared to PV blocks (further referred to as ‘*working memory (WM) maintenance costs*’) represent the sustained processing dynamics underlying the stable maintenance of information in the face of external interference. DS as compared to IS blocks (further referred to as ‘*WM scheduling costs*’) represent the sustained processing dynamics underlying the coordination and scheduling of dual task sets.

Participants completed three practice blocks of 12 trials each, one containing DS trials, one containing only IS trials, and one containing only PV trials. Afterward, participants performed through six experimental blocks (each condition twice), where each block consisted of 20 trials, with ten matching and ten non-matching cue-probe combinations (or left- and right-pointing arrow in the PV condition) in random order. In the IS condition, two extra trials per block were added to replace the 10% of catch IS trials that were discarded from the analyses. Response assignments were counterbalanced across participants, and so was the order of conditions. The same tasks were used at pre- and posttest, but with different stimulus sets. Each task block was framed by two fixation blocks of 32.4 s each. The total duration of the task amounted to approximately 30 min (excluding practice blocks, instruction periods, and breaks), and was split into six scanning runs.

Again, trial events were synced to the repetition time (TR) of two successive scanner pulses (see also Figure 3B). Each trial started with the presentation of a fixation cross for 200 ms, followed by the cue landscape for 800 ms and a first delay period (2,600 ms), adding up together to 2 TR. Subsequently, the interfering face stimulus was presented for 800 ms, and followed by a second delay of 2,800 ms (again amounting to 2 TR). Then, the probe stimulus (landscape or arrow) appeared on the screen for 1,800 ms (1 TR). Participant’s responses were again recorded up to a maximum of 2 TR after probe onset. If participants responded faster than 1 TR, a blank was inserted that filled up the time until the next scanner pulse was applied (1 TR minus individual response time). The inter-trial interval (ITI) was presented variably, ranging between 1 and 3 TR. Our ITI durations followed again a geometric distribution (Ashby, 2011). At the end of each block, participants received feedback about their mean reaction time (RT) and the achieved number of correct responses. Participants were asked to respond to the probe stimulus as quickly and as accurately as possible with their index fingers.

### Apparatus and fMRI Acquisition

The computer experiments for both pre- and posttest-assessment and training sessions were programed via E-Prime^®^ 2.0 Professional (Psychological Software Tools, 2012).

Imaging data were collected on a 3 Tesla MRI system (MAGNETOM Skyra, Siemens Healthcare) with a 20-channel head coil. Visual stimuli were presented via a projector screen, which was placed at the top end of the scanner bore. A mirror on the top of the head coil enabled the participants to view the screen. Earplugs and headphones dampened the scanner noise but still enabled communication with the participants. Manual responses were recorded via NordicNeuroLab (NNL) fiber optic response grips. Trial event onsets were synced to the scanner trigger pulse by using the NNL SyncBox. The protocol of each session (pretest, posttest) included localizer images, low- and high-resolution T1-weighted structural images, a series of T2^∗^-weighted functional images, and diffusion tensor imaging (DTI) sequences. In the present study, we only examined T1-weighted structural and T2^∗^-weighted functional images. The high-resolution anatomical scans were acquired using a sagittal MP-RAGE T1-weighted sequence (192 slices, slice thickness = 0.9 mm, in-plane resolution = 0.938 × 0.938 mm^2^, repetition time (TR) = 1,900 ms, echo time (TE) = 2.13 ms, inversion time (TI) = 900 ms, flip angle = 9n°, field of view = 240 × 240 mm^2^). Whole brain functional images were collected in sequential (ascending) order in 32 axial slices, co-planar with the anterior-posterior commissure (AC-PC line), with a thickness of 3 mm based on T2^∗^-weighted gradient echo-planar imaging (EPI) sequences with the following parameters: TR = 1,800 ms, TE = 30 ms, flip angle = 90°, inter-slice gap = 0.75 mm, field of view = 192 × 192 mm^2^, matrix size = 94 × 94, voxel size = 3 × 3 × 3 mm^3^, parallel-imaging method: GRAPPA.

### Customized Longitudinal fMRI Data Preprocessing Pipeline

fMRI preprocessing and data analyses were carried out using the statistical parametric mapping software (SPM 12; Wellcome Trust Centre for Neuroimaging, Institute of Neurology, University College London, United Kingdom^7^), on MATLAB R2014a (MathWorks, Natick, Massachusetts, United States), in combination with the computation anatomy toolbox extension^8^ (CAT12). Our preprocessing pipeline included steps for head motion and slice timing correction as well as co-registration, normalization, and smoothing steps. Importantly, we implemented a customized longitudinal preprocessing pipeline to account for our multi-session design. For details, see Supplementary Material SI1.

### First-Level General-Linear Model Approach

Regarding the *transient-sensitive context-updating task*, we first accounted for low-frequency signal changes and baseline drifts by high-pass filtering the data at 0.004 Hz. A general-linear model (GLM) approach was applied to estimate parameters for event-related (transient) effects.

For each condition type (i.e., c-dep, c-indep) of each run (1,2) in each session (pretest, posttest), we created a primary regressor of interest based on the respective trial onsets and a zero duration. To control for time-on-task differences between conditions that were of no interest for the present study, for each condition, we included a parametric regressor in our statistical model that was defined by the same onsets as the primary regressors but by a duration varying from trial to trial according to response times. Each of these parametric modulators (PM) was orthogonalized with respect to the primary regressor within event type. Transient effects were estimated by the time-course of event-related responses for each condition without an assumed shape of the HRF, using a series of 13 time bins along a hemodynamic response epoch taken to be 23.4 s (Braver et al., 2003, 2009; Jimura and Braver, 2009).

A set of covariates of no interest was included in our statistical model; that is, practice trials, error trials, instruction periods preceding each task block, and feedback periods following each task block. Moreover, we accounted for the six movement regressors. Baseline activation levels were estimated as an average of all residual time points (implicit baseline).

Regarding the *sustained-sensitive delayed-recognition task*, we estimated parameters for block-related (sustained) effects again based on a general-linear model (GLM) approach. Sustained effects were coded by a single regressor for each block type (DS, IS, PV) of each run (1 to 6) and session (pretest, posttest), modeled as a boxcar function convolved with SPM’s canonical double-gamma HRF, with onsets corresponding to the trial onset of the first experimental trial per block, and durations corresponding to the task-block length.

The same set of covariates of no interest as above was included in our statistical model; that is, practice trials, error trials, instruction periods preceding each task block, and feedback periods following each task block. We further included the slope of a linear drift within each run in place of a high-pass filter as such filtering might attenuate the relatively low-frequency sustained effects (cf. Braver et al., 2003; Reynolds et al., 2008; Jimura and Braver, 2009; Oksanen et al., 2014). Moreover, we controlled again for the six movement regressors. Baseline activation levels were estimated as average of all residual time points.

### ROI Analysis

#### ROI Selection

**FIGURE 4:**
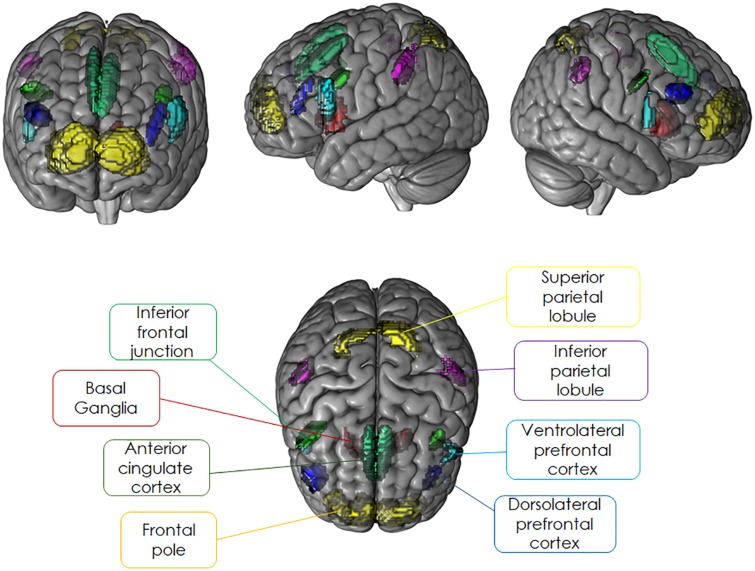
Literature-based probabilistic regions of interest overlaid as colored maps onto template brain (coronal, sagittal, and horizontal planes).

For the present study, we restricted our analyses to brain regions belonging to cognitive control regions that have been shown to be sensitive to the modulation of sustained or transient activation dynamics and were identical to the ROIs selected for our previous study (Dörrenbächer et al., 2017a,b). Independent *a priori* ROIs allowing for small volume alpha error adjustment were created combining anatomical hypotheses with functional findings as reported in literature for experimental designs comparable to the task-switching training paradigm (see Figure 4 and Table 2). For details, see Supplementary Material SI2.

**TABLE 2:** Overview of literature-based probabilistic regions of interest used to mask the neuroimaging data.

	**ROI**	**Included BA**	**Hemi**	**Center of mass (MNI coordinates)**			**Volume (cm^3^)**
				***x***	***y***	***z***	
Transient-sensitive task-switching inclusion mask	Mid-VlPFC	44,45	L	−47	17	22	2.58
			R	46	15	15	0.92
	IFJ	6,8,9,44 (intersection)	L	−43	7	34	1.31
			R	46	4	35	1.97
	SPL	7	L	−18	−64	51	3.37
Sustained-sensitive task-switching inclusion mask	BG (putamen, caudate nucleus)	n. a.	L	−14	7	−2	3.53
			R	14	9	−4	2.38
Task-switching exclusion mask	FP	10,11	L	−26	54	8	12.72
			R	26	55	13	15.09
	ACC	6,8,32	L	−4	17	44	4.34
			R	4	17	47	5.88
	DlPFC	9,46	L	−39	33	20	1.57
			R	44	33	27	2.63
	SPL		R	18	−61	54	2.06
	IPL	40	L	−45	−47	46	2.65
			R	44	−45	44	1.44

*ROI = Region of interest; BA = Brodmann area; Hemi = hemisphere; FP = frontal pole; ACC = anterior cingulate cortex; BG = basal ganglia; DLPFC = dorsolateral prefrontal cortex; Mid-VLPFC = ventrolateral prefrontal cortex; IFJ = inferior frontal junction; SPL = superior parietal lobule; IPL = inferior parietal lobule; L = left; R = right.*

#### Extraction of Summary Measures

Using the SPM toolbox extension MarsBaR v0.44 (Brett et al., 2002), we extracted the individual first-level parameter estimates for each effect of interest (i.e., respective contrasts of condition of interest versus implicit baseline) averaged across all selected voxels falling anywhere within each ROI mask (Poldrack et al., 2011). All effects were estimated as percent signal change, which was calculated as the magnitude of the parameter estimate for a condition of interest, scaled by the maximum value of a reference trial or reference block of the same duration computed at the resolution of the super-sampled design matrix, divided by the mean estimate for baseline activation per each run in each session, and multiplied by 100 (Pernet, 2014). Percent-change transformed data were averaged across runs, and then entered into group-level analyses.

#### Data Analysis

For all analyses, contrast *t*-values for planned comparisons were transformed into *F*-values with one numerator degree of freedom (df) and reported together with one-tail *p*-values.

#### Behavioral Analyses

Analyses of behavioral data were presented for both the proportion of errors (ER,%) as well as latencies for correct responses (RT, ms). Practice blocks and the first trial of each block were excluded from data analysis. For all tasks, trials with latencies faster than 200 ms and slower than 3,600 ms were treated as misses and added to error rates. To investigate training-group differences in the slope of improvement, we conducted a three-way ANOVA, including the between-subjects factor training group (TG, CG), and the within-subjects factors session (pretest, posttest), and condition (c-dep, c-indep) or block type (DS, IS, PV).

The block type factor for the delayed-recognition task was further specified by two orthogonal contrasts: *WM maintenance costs* were calculated as the difference in mean performance in DS plus IS blocks against baseline PV blocks (contrast: 1 1 −2) and *WM scheduling costs* were calculated as the difference in performance in DS against IS blocks (contrast: −1 1 0).

#### Neuroimaging Analyses

*P*-values for neuroimaging analyses were corrected for Type-I-error accumulation for the number of tested ROIs within each mask image using Holm’s sequential Bonferroni correction method (Holm, 1979).

##### Training-group differences in transient brain activation dynamics (context-updating task)

A first set of analyses focused on training-group differences in *transient* brain activation dynamics. Region-wise percent signal changes in BOLD responses (%) of the transient regressors were subjected to a three-way ANOVA, including the between-subjects factor training group (CG, TG), session (pretest, posttest), and trial type (c-dep, c-indep). We were specifically interested in ROIs showing sensitivity to a group-selective shift in the magnitude of transient task-related activation from pre- to posttest. The criterion for such group-selective neural changes was set conservatively:

(i) to a significant *group* × *session* × *trial type* crossover interaction testing whether groups would differ in changes in the magnitude of *context-updating costs activation*;
(ii) and, by examining the interaction, a significant change in the transient context-updating costs activation at posttest relative to pretest *that was greater in the TG than in the CG and/or exclusively found for the TG* and was associated with behavioral improvement.

##### Training-group differences in sustained brain activation dynamics (delayed-recognition task)

A second set of analyses focused on training-group differences in *sustained* brain activation dynamics. Region-wise percent signal changes in BOLD responses (%) of the block regressors were subjected to a three-way ANOVA, including the between-subjects factor training group (CG, TG), session (pretest, posttest), and block type (DS, IS, PV). Again, we were specifically interested in ROIs that were sensitive:

(i) to a significant *group* × *session* × *block type* crossover interaction testing whether groups would differ in changes in the magnitude of *WM maintenance costs activation* and/or *WM scheduling costs activation* in the TG as compared to the CG;
(ii) by examining the interaction, a significant change in sustained brain activation at posttest relative to pretest *that was greater in the TG than in the CG and/or exclusively found for the TG* and was associated with behavioral improvement.

##### Neural overlap of training group differences in trained and untrained tasks

Importantly, for both transient (i.e., context-updating task) and sustained changes (i.e., delayed-recognition task), we further aimed to examine whether any selective changes would be based on spatial, temporal, or spatio-temporal overlap with activation changes found previously in the task-switching training task (cf. Dörrenbächer et al., 2017a,b). To test for neural correlates of selective changes in the untrained task that were overlapping with task-switching training effects, we computed three spatial mask images:

(i) a *transient-sensitive task switching inclusion mask* image that only included ROIs that had been sensitive to changes in transient activation in the training task (i.e., left and right ventrolateral PFC, left and right inferior frontal junction, left superior parietal lobule).
(ii) a *sustained-sensitive task switching inclusion mask* image that only included ROIs that had been sensitive to changes in sustained activation in the training task (i.e., left and right basal ganglia).
(iii) a *task switching exclusion mask* image that included canonical ROIs outside those obtained for the task-switching training task (i.e., frontal pole, dorsolateral prefrontal cortex, anterior cingulate, inferior parietal lobule).

For each of these spatial masks, we ran our analyses for training-group differences in changes in transient or sustained brain activation dynamics separately. *Only spatial overlap* was defined as group-selective changes found for the *context-updating task* in ROIs within the *sustained-sensitive inclusion mask*, or for the *delayed-recognition task* in ROIs within the *transient-sensitive inclusion mask*.

*Only temporal overlap* was defined as group-selective changes found for the *context-updating or the delayed-recognition task* in rois within the *exclusion mask*.

*Spatio-temporal overlap* was defined as group-selective changes found for the *context-updating task* in ROIs within the *transient-inclusion mask* or for the *delayed-recognition task* in ROIs within the *sustained-inclusion mask*.

## Results

### Training-Group Differences in Behavioral Changes

**FIGURE 5:**
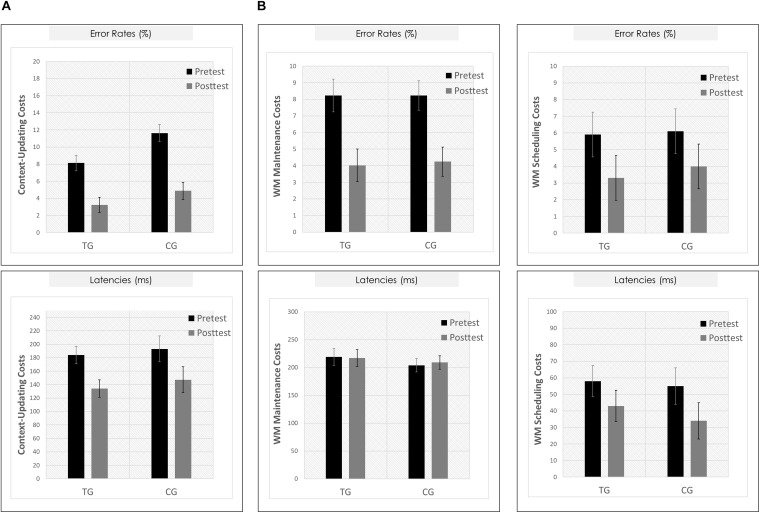
Behavioral results. **(A)** Context-updating paradigm. *Upper Panel*: Mean updating costs on error rates (absolute values,%); *Lower Panel*: Mean updating costs on reaction times (absolute values, ms); each as a function of Session (pretest/black bar, posttest/gray bar) and Group (TG/the two bars on the left; CG/the two bars on the right). **(B)** Delayed-recognition paradigm. *Upper Row*: Mean costs on error rates (absolute values,%); *Lower Row*: Mean costs on reaction times (absolute values, ms); *Left Panels*: Working-memory maintenance costs; *Right Panels*: Working-memory scheduling costs; each as a function of Session (pretest/black bar, posttest/gray bar) and Group (TG/the two bars on the left; CG/the two bars on the right). ^∗^*p* ≤ 0.05. TG = task-switching training group; CG = active control group. Error bars depict standard errors of the mean (SEM) based on the session × condition interaction within each group comparing within-subjects session conditions according to Jarmasz and Hollands (2009). Note that the selected variance estimators are not suited to directly compare group conditions. Please also note that for display purposes, we plotted here the absolute values of costs in order to emphasize differences in magnitude, irrespectively of directional changes within condition. However, raw values can be found in Table 3.

Means and SDs for all groups as a function of condition (c-indep, c-dep) or block type (DS, IS, PV) and session (pretest, posttest) are presented in Table 3. For the context-updating task (see Figure 5A), results revealed a significant session × condition interaction on error rates (*F*(1,42) = 22.77, *p* = 0.00, η_*p*_^2^ = 0.35) as well as on latencies (*F*(1,42) = 4.45, *p* = 0.04, η_*p*_^2^ = 0.10), suggesting a reduction of updating costs from pretest to posttest. However, the interaction with group as factor did not reach significance (ER: *p* = 0.21, RT: *p* = 0.45).

**TABLE 3:** Means and standard deviations for behavioral data (reaction times, error rates) as a function of training group (single-task active control group/CG, task-switching training group/TG), task (context-updating task, delayed-recognition task), session (pretest, posttest), and condition (context-updating task: context-independent/c-indep, context-dependent/c-dep; delayed-recognition task: passive viewing/PV, distraction/DS, interruption/IS).

			**Group**			
			**Single-task active control group (CG)**		**Task-switching training group (TG)**	
			***M***	***SD***	***M***	***SD***
**Context-updating task**						
Reaction times (ms)						
	Pretest	c-indep	699	127	611	113
		c-dep	887	228	795	158
	Posttest	c-indep	549	92	525	74
		c-dep	693	127	658	143
Error rates (%)						
	Pretest	c-indep	1.9	2.8	1.3	2.0
		c-dep	13.5	11.0	9.4	8.5
	Posttest	c-indep	1.0	1.5	0.7	1.7
		c-dep	5.1	5.8	3.5	4.0
**Delayed-recognition task**						
Reaction times (ms)						
	Pretest	PV	550	70	541	87
		DS	736	121	744	141
		IS	771	123	776	140
	Posttest	PV	495	66	472	68
		DS	691	91	672	126
		IS	718	100	706	131
Error rates (%)						
	Pretest	PV	0.6	1.3	0.7	1.3
		DS	7.4	6.8	7.9	8.2
		IS	10.3	6.4	10.0	7.2
	Posttest	PV	1.0	1.9	0.7	1.3
		DS	4.4	4.6	3.6	3.4
		IS	5.9	3.2	5.8	4.2

*M = mean; SD = standard deviation.*

For the delayed-recognition task (see Figure 5B), we obtained a similar pattern of results: We found significant changes in behavioral performance from pre- to posttest on the level of error rates (*F*(2,80) = 8.02, *p* = 0.00, η_*p*_^2^ = 0.17; however, not on the level of latencies, *p* = 0.47) indicating a decrease of WM maintenance error costs (*F*(1,40) = 20.05, *p* = 0.00, η_*p*_^2^ = 0.33), albeit not of WM scheduling error costs (*p* = 0.31). Again, behavioral improvement was not modulated by the training condition (ER: *p* = 0.40; RT: *p* = 0.42).

### Neuroimaging Data

Means and SDs for all groups as a function of condition (c-indep, c-dep) or block type (DS, IS, PV) and session (pretest, posttest) are presented in Table 4.

**TABLE 4:** Means and standard deviations for neural data (percent signal change) as a function of training group (single-task active control group/CG, task-switching training group/TG), region of interest (left inferior parietal lobule, right basal ganglia), session (pretest, posttest), and condition (context-updating task: context-independent/c-indep, context-dependent/c-dep; delayed-recognition task: passive viewing/PV, distraction/DS, interruption/IS).

			**Group**			
			**Single-task active control group (CG)**		**Task-switching training group (TG)**	
Changes in transient dynamics (left inferior parietal lobule)			***M***	***SD***	***M***	***SD***
			
	Pretest	c-indep	0.019	0.231	0.099	0.134
		c-dep	0.088	0.178	0.031	0.177
	Posttest	c-indep	0.059	0.126	0.015	0.176
		c-dep	0.030	0.181	0.009	0.204
Changes sustained dynamics (right basal ganglia)						
	Pretest	PV	0.029	0.352	−0.004	0.360
		DS	0.271	0.567	−0.150	0.496
		IS	0.132	0.439	0.173	0.498
	Posttest	PV	−0.131	0.862	−0.445	1.403
		DS	−0.075	0.433	0.191	0.687
		IS	0.018	0.326	0.140	0.389

*M = mean; SD = standard deviation.*

#### Training-Group Differences in Transient Brain Activation Dynamics (Context-Updating Task)

For the context-updating task, we found a significant group × session × trial type crossover interaction within *the task switching exclusion mask*. More specifically, on transient brain activation in the left site of the dorsolateral PFC (only by tendency: *F*(1,42) = 2.74, *p* = 0.07, η_*p*_^2^ = 0.06) as well as in the left site of the inferior parietal lobule (*F*(1,42) = 4.01, *p* = 0.03, η_*p*_^2^ = 0.09). We did not find group-differential changes of the transient context-updating costs activation in any ROI within the two *task switching inclusion masks* (all *p’s* > 0.07), except for the right inferior frontal junction (*F*(1,42) = 4.42, *p* = 0.04, η_*p*_^2^ = 0.10, yet pointing to the opposite direction, with the CG showing larger effects; see Supplementary Materials SI3, SI5, and SI6 for details about all region-wise results).

**FIGURE 6:**
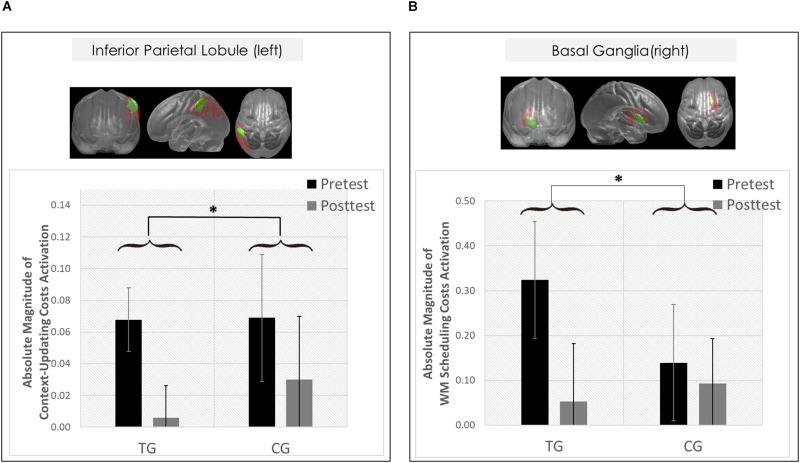
Neural results. **(A)** Changes in *transient* brain dynamics (i.e., context-updating paradigm, absolute magnitude of context-updating costs activation) in the left inferior parietal lobule. Mean updating-costs activation in percent signal change units (absolute values,%) as a function of Session (pretest/black bar, posttest/gray bar) and Group (TG/the two bars on the left; CG/the two bars on the right). **(B)** Changes in sustained brain dynamics (i.e., delayed-recognition paradigm, absolute magnitude of WM scheduling costs activation) in the right basal ganglia as a function of Session (pretest/black bar, posttest/gray bar) and Group (TG/the two bars on the left; CG/the two bars on the right). ^∗^*p* ≤ 0.05. TG = task-switching training group; CG = active control group. Error bars depict standard errors of the mean (SEM) based on the session × condition interaction within each group comparing within-subjects session conditions according to Jarmasz and Hollands (2009). Note that the selected variance estimators are not suited to directly compare group conditions. Please also note that for display purposes, we plotted here the absolute values of costs in order to emphasize differences in magnitude, irrespectively of directional changes within condition. However, raw values can be found in Table 4.

**FIGURE 7:**
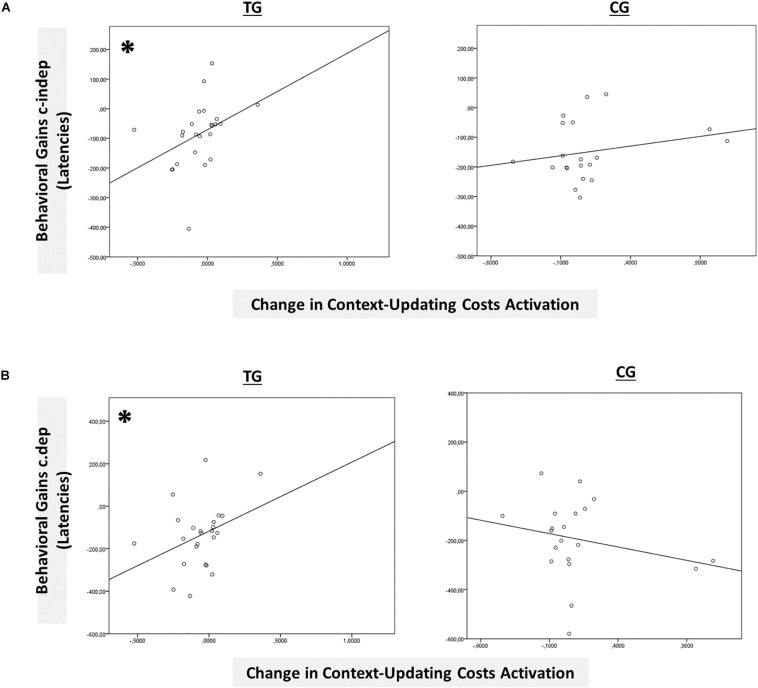
Brain-behavior relationships. **(A)** Group-wise brain-behavior relationships between pretest-posttest change in transient context-updating costs activation in the IPL and behavioral gains in c-indep trials (latencies). **(B)** Group-wise brain-behavior relationships between pretest-posttest change in transient context-updating costs activation in the IPL and behavioral gains in c-dep trials (latencies). ^∗^*p* ≤ 0.05. TG = task-switching training group; CG = active control group.

However, only in the left inferior parietal lobule within the *exclusion mask*, changes in the magnitude of context-updating costs activation from pre- to post-test reached significance only in the TG (*F*(1,42) = 3.33, *p* = 0.04, η_*p*_^2^ = 0.13), while not in the CG (*p* = 0.11), as can also be seen in Figure 6A (please note that for display purposes, we plotted the absolute values of costs in order to emphasize differences in magnitude, irrespectively of directional changes within condition). It should be noted that groups did not differ in the magnitude of context-updating costs activation in the left inferior parietal lobule at pretest (*p* = 0.15), ruling out the influence of preexisting differences in baseline activation. Importantly, the pre-post change in the magnitude of context-updating costs activation in the left inferior parietal lobe was associated with behavioral improvement at the latency level both in c-indep trial conditions (*r*(22) = 0.38, *p* = 0.03) as well as in c-dep trial conditions (*r*(22) = 0.36, *p* = 0.04) within the TG, while not within the CG (c-indep: *r*(18) = 0.23, *p* = 0.17; c-dep: *r*(18) = −0.24, *p* = 0.16, see Figures 7A,B). However, no correlations between activation changes and behavioral performance gains were found within each group at the error level (all *p*’s > 0.36).

Hence, only the left inferior parietal lobule (within the *exclusion mask*) met the criteria of a group-selective shift as defined in the section “Data Analysis,” pointing to *temporal but no spatial overlap* of transient changes in the context-updating task with the task-switching training task.

#### Training-Group Differences in Sustained Brain Activation Dynamics (Delayed-Recognition Task)

For the delayed-recognition task, we found a significant group × session × block type crossover interaction on sustained activation within the *sustained-sensitive task switching inclusion mask*, namely in the left (*F*(2,80) = 4.68, *p* = 0.00, η_*p*_^2^ = 0.11) and right site of the basal ganglia (*F*(2,80) = 2.78, *p* = 0.04, η_*p*_^2^ = 0.07). We found no group-differential changes of sustained WM-scheduling costs brain activation in any other ROI within the *transient-sensitive inclusion mask* or within the *exclusion mask*, nor of sustained WM-maintenance costs brain activation in general (all *p’s* > 0.13), except for the right inferior parietal lobule (WM-scheduling costs activation: *F*(1,40) = 4.80, *p* = 0.03, η_*p*_^2^ = 0.11) and the left superior parietal lobule (WM-maintenance costs activation: *F*(1,40) = 3.18, *p* = 0.02, η_*p*_^2^ = 0.07; see Supplementary Materials SI4, SI5, SI7 for details about all region-wise results).

**FIGURE 8:**
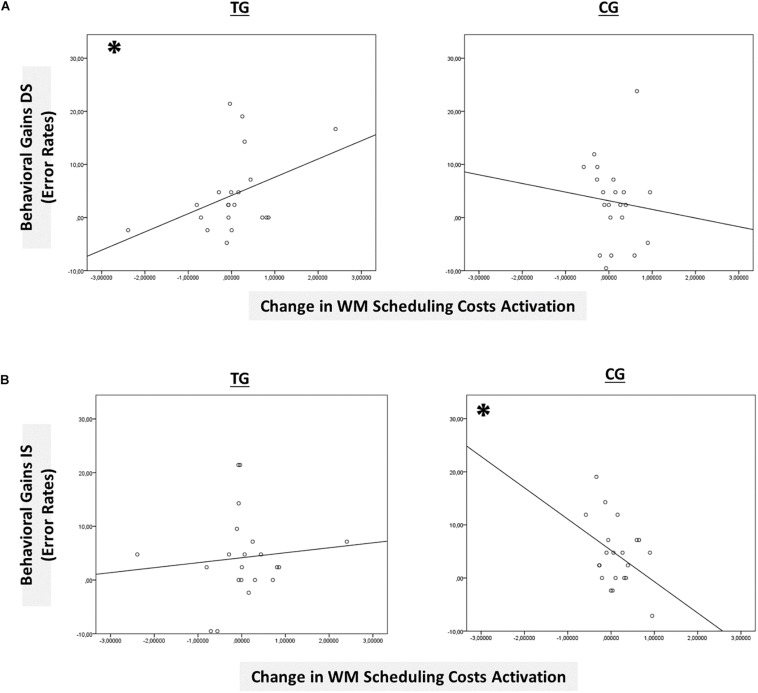
Brain-behavior relationships. **(A)** Group-wise brain-behavior relationships between pretest-posttest change in sustained WM scheduling costs activation in the basal ganglia and behavioral gains in the distracter condition (error rates). **(B)** Group-wise brain-behavior relationships between pretest-posttest change in sustained WM scheduling costs activation in the basal ganglia and behavioral gains in the interrupter condition (error rates). ^∗^*p* ≤ 0.05. TG = task-switching training group; CG = active control group. DS = distracter condition. IS = interrupter condition.

However, only in the right site of the basal ganglia within the *sustained-sensitive task switching inclusion mask*, the session × block type interaction reached significance in the TG (*F*(2,40) = 1.61, *p* = 0.05, η_*p*_^2^ = 0.11), while not in the CG (*p* = 0.12). More specifically, in the right basal ganglia, the TG showed a change in the magnitude of WM-scheduling costs activation from pre- to posttest (*F*(1,20) = 2.86, *p* = 0.05, η_*p*_^2^ = 0.13), as can also be seen in Figure 6B (please note again that for display purposes, we plotted here the absolute values of costs in order to emphasize differences in magnitude, irrespectively of directional changes within condition), while not of WM-maintenance costs activation (*p* = 0.08; see Table 4 and Supplementary Material SI7 for details). Groups did not differ in the magnitude of WM-scheduling costs activation in the right basal ganglia at pretest (*p* = 0.15), pointing to robust training-induced changes. Importantly, the pre-post change in the magnitude of WM-scheduling costs activation in the right basal ganglia was associated specifically with behavioral gains at the accuracy level in distracter blocks (*r*(19) = 0.41, *p* = 0.03) within the TG, while not within the CG (DS: *r*(19) = −0.09, *p* = 0.36, see Figure 8A). Although the TG showed no brain-behavior-relationship in interrupter blocks (IS: *r*(19) = 0.10, *p* = 0.33), the CG did so, yet pointing to the opposite direction (IS: *r*(19) = −0.39, *p* = 0.04) suggesting rather task-adverse effects (i.e., the larger the magnitude of brain-activation changes, the smaller performance gains, see Figure 8B). No group-selective brain-behavior relationships were found at the level of latencies (all *p*’s > 0.27).

Hence, only the right basal ganglia (within the *sustained-sensitive inclusion mask*) met our criteria of a group-selective shift as defined in the section “Data Analysis,” pointing to *spatio-temporal overlap* of sustained changes in the delayed-recognition task with the task-switching training task.

## Discussion

The aims of the present study were, first, to examine neural plasticity after task-switching training (as compared to active-control single-task training) beyond behavioral changes by considering both the spatial location and the timescale of brain activation changes; and second, to provide evidence for spatio-temporal dynamics of neural changes in untrained cognitive tasks.

### Training-Group Differences in Behavioral Changes

Behavioral data suggested training-induced changes in both context-updating and WM-maintenance costs for both training conditions. However, results did not reveal robust training-group differences in behavioral improvements, raising some doubts on the generality of benefits from such training (for a review, see also Simons et al., 2016). The net behavioral transfer gains due to executive-control training in older adults are assumed to be small, if present at all (for a meta-analysis, see Karbach and Verhaeghen, 2014). The controversy is even reflected in two previous training studies from our own lab: in one of our first task-switching training studies (Karbach and Kray, 2009), we found indeed selective changes in measures of inhibition or working memory in older adults after task switching as compared to single-task training with medium to large effect sizes. However, in another recent study (Kray and Fehér, 2017), we did not find evidence for transfer to untrained cognitive-control tasks. Some specific variations in design features, such as the type of the involved stimuli and training tasks (i.e., differing degrees of variability, predictability, induced interference) may be important here. Compared to these former training regimes, the present study applied some other design variations (i.e., an unpredictable, cued paradigm variant coupled with a response deadline) to make the paradigm more suitable to the particular characteristics of our neural measurements, which may have contributed to the small training-group differences at the behavioral level. In addition, our single-task active control group had been designed in a very conservative fashion (see section “Materials and Methods” for details): first, it included the same amount of training variability across sessions as the task-switching training group. Second, within each single-task training session, participants had to alternate between single-task blocks of the two relevant tasks, thus this condition also put demands on shifting, albeit with a lower frequency (i.e., at the block level). Third, our single-task group had also experienced some training of inhibitory control by practicing with the same ambiguous stimulus material with overlapping response formats.

Moreover, as outlined in the section “Introduction,” the training results from our study that are presented in detail elsewhere (Dörrenbächer et al., 2017a,b) have already suggested small training group differences at the behavioral level. In that study, by examining the neural mechanisms, however, we uncovered more subtle training-induced effects beyond behavioral change, a pattern that also appears to apply to the effects in the present study. Alternatively, we would like to draw attention to the fact that our training groups had both received a relatively extensive amount of practice, that is, double the number of training sessions as in Karbach and Kray (2009) or Kray and Fehér (2017). Hence, in the present study, we may have isolated neural differences at the boundaries of potential cognitive plasticity in our task switching paradigm variant (see also Baltes et al., 1999).

### Training-Group Differences in Changes in Transient Brain Activation Dynamics (Context-Updating Task)

On the level of *transient* neural dynamics, we found training-group differences in changes of the context-updating costs activation in some prefrontal and parietal regions, yet only being selective for the task-switching training group in the left inferior parietal lobule. The inferior parietal cortices have shown consistent involvement in executive processes of working memory (Nee et al., 2013). More specifically, the inferior parietal lobe is involved across several variants of context-updating tasks, thereby supporting functions, such as the online maintenance (Lopez-Garcia et al., 2016) and updating of working-memory content (Nee and Brown, 2013) as well as the temporal organization of control processes (Lesh et al., 2013). In the present study, after task switching compared to single-task training, participants needed less recruitment of this parietal area to update the task context, as reflected in the difference between c-indep and c-dep trial conditions, which seemed to facilitate performance. A similar result was obtained by Heinzel et al. (2016) after WM training in older adults. The authors revealed a selective decrease of BOLD signals in canonical WM networks in the n-back task for their older adult training group compared to passive controls. This was attributed to a training-related boost of processing efficiency.

In the present study, we were specifically interested in the change in *magnitude* of brain activation (i.e., the change in the overall amount of neural energy invested into the task), while the direction of changes (i.e., an up- or down-regulation of activation) was of lesser importance. Yet, the differential directional changes of activation in both training groups, especially within trial conditions, may crucially affect the interpretation of data. Given the different directions of brain-activation changes within condition between TG and CG – more specifically, an activation decrease in both c-dep and c-indep trials in the TG, but an activation decrease in c-dep trials and an increase in c-indep trials in the CG (see Table 4 containing the raw values) – may also point to qualitative (e.g., strategy) differences. Brain-behavior relationships indicated that only the neural pattern of the TG seemed to promote better behavioral performance within condition. Their pattern fits to research indicating that high-performing (i.e., better-trained) people tend to enhance their overall neural efficiency (Neubauer and Fink, 2009) and that older adults specifically recruit similar neural resources for various task conditions in order to enable a non-selective processing style (DiGirolamo et al., 2001; Karayanidis et al., 2010; Schmitt et al., 2014; Whitson et al., 2014). However, as described in the section “Introduction,” the direction of neural change associated with optimal task processing is still a matter of debate (e.g., the neural-efficiency versus the cortical-effort hypothesis, see Barulli and Stern, 2013). Therefore, future research should further elucidate the specificity of (ability- and practice-dependent) adaptions of brain activation to the respective task demands (see also Dunst et al., 2014).

Moreover, our result pattern could also be influenced by pretest. Yet, we found no substantial group differences in context-updating costs activation at pretest. Nevertheless, future research should address the issue of baseline differences more thoroughly, for example, by *a priori* matching participants to the different training regimes according to their pretest neural activation patterns.

Comparing the change pattern for the context-updating task with the pattern of transient activation changes found for the task-switching training task in Dörrenbächer et al. (2017a,b), we found training-induced changes in a region *outside those obtained for the task-switching training task*, indicating *only temporal overlap*. This result suggests that our task-switching training may have indeed fostered better temporal organization of control processing in another control task, which will be discussed in more detail below.

### Training-Group Differences in Changes in Sustained Brain Activation Dynamics (Delayed-Recognition Task)

On the level of sustained neural dynamics, we found group differences in the amount of reducing WM-scheduling costs activation in the right basal ganglia. The obtained neural change effects for the scheduling costs activation, but not for the maintenance costs activation, suggest that the changes were specifically related to the scheduling component of training. Clapp et al. (2010) showed that indeed, distinct neural mechanisms may mediate the impact of different types of external interference on WM (i.e., distraction versus interruption) in aging. The selective effects on WM scheduling mechanisms seems plausible given that the interruption condition with a secondary task, as compared to the distraction condition, put additional demands on the coordination and dynamic shift between different task-sets (i.e., multi-tasking), which is also a key process in the task switching paradigm and may lie at the core of aging effects therein (Clapp et al., 2010, 2011). At the neural level, Clapp et al. (2011) point to an ‘interruption recovery failure, manifest as a deficient ability to dynamically switch between functional brain networks’ (p. 7212). However, group-selective relationships of brain-activation changes were specifically found for behavioral improvement in the distracter condition, and not in the interrupter condition. Therefore, further research should disentangle both components in more detail.

The striatal BOLD response has been frequently defined as a direct proxy for DA release in the basal ganglia (Schott et al., 2008). A number of studies linked practice in executive-control tasks to changes in the basal ganglia and striatal DA signaling, where an increase in activation seemed to mediate learning or training-related improvements in WM (Kühn et al., 2013; Salminen et al., 2016), specifically in WM updating (e.g., Westerberg et al., 2007; Jolles et al., 2010; Salminen et al., 2016). For example, Dahlin et al. (2008) found in younger adults a training-induced increase of brain activation in the striatum, reflecting increased involvement of task-specific updating processes required in a dual *n*-back task (i.e., ‘specific process-improvement,’ cf. Salminen et al., 2016), beyond a training-induced decrease of brain activation in the frontoparietal network, implying less requirement of general cognitive processes with better task performance (i.e., ‘general boosting,’ cf. Salminen et al., 2016). Similarly, Salminen et al. (2016) revealed an increase in striatal activation that was linked to training-related strengthening of those neural resources that support successful task-specific updating processes (i.e., ‘specific process-improvement,’ Salminen et al., 2016).

While transient striatal dopamine signaling has indeed been linked to such gating and updating mechanisms, sustained DA release in the basal ganglia has been primarily associated with WM maintenance and general responsivity (Cohen et al., 2002; Braver et al., 2005; Murty et al., 2011). Older adults show pronounced reductions in amplitudes of *sustained DA activation* in the basal ganglia (Braver et al., 2005), where such alterations have been attributed to unspecific losses in sustained activation resources (Braver et al., 2003; Dennis et al., 2007). Hence, an important new insight of our study was the capture of training-related changes in *sustained* activation in the basal ganglia after task-switching training in older adults, which was associated with improved WM maintenance performance.

The neural alterations in older adults may also fundamentally disrupt the *homeostasis between sustained and transient activation levels* in striatal loops, resulting in less flexible recruitment of sustained activation resources, where transient activation mechanisms appear largely preserved but are recruited in a different fashion (Jimura and Braver, 2009). Hence, it could be also an important avenue for older adults to rely more heavily on still-preserved neural mechanisms (i.e., transient activation) as a means to compensate for losses in sustained resources (Jimura and Braver, 2009). Therefore, future training studies in older adults should directly compare changes in sustained and transient processes, preferably within the same analysis, such as based on mixed epoch-/event-related experimental designs (e.g., Braver et al., 2003; Jimura and Braver, 2009; for a general review, see Visscher et al., 2003, see also the section “Further Limitations and Outlook”). Future studies should further complement findings by molecular imaging techniques to capture DA signaling more directly.

Comparing our results pattern with the pattern of sustained activation dynamics found for the task-switching training task (see Dörrenbächer et al., 2017a,b), the training-induced sustained changes overlapped in both region and timescale with the task-switching training task, indicating *spatio-temporal overlap*. This result indicates that task-switching training may engage very similar neural mechanisms in another control task that also taps into sustained control of different task sets. This will be further discussed in the next section.

### Spatio-Temporal Interactions in Neural Changes

Summarizing the neural results, analyses suggest that neural changes were supported by spatio-temporal interactions, similarly to the training results in our previous study (Dörrenbächer et al., 2017a,b). More specifically, for the sustained-sensitive delayed-recognition task, we obtained group-selective changes in sustained activation in exactly the same region where we had previously found training-induced changes in the task switching task (i.e., in the basal ganglia). Hence, changes in the sustained-sensitive task might be clearly overlapping in both spatial and temporal features of brain activation with the training task. However, for the transient-sensitive context-updating task, we obtained selective changes in transient activation in different fronto-parietal regions than those where we had previously found training-induced changes in the task switching task (i.e., in the right dorsolateral PFC and in the inferior parietal lobule). Hence, these changes cannot be simply traced back to overlapping spatial features of brain activation in the trained and untrained task (Dahlin et al., 2008) but potentially point to temporal overlap.

These results suggest that neural changes in untrained cognitive tasks may indeed presume an overlap in ‘neural resources’ (Karbach and Kray, 2016) but these neural resources may not be necessarily defined only spatially (Dahlin et al., 2008), but can also be defined temporally, especially in the context of executive-control training. This opts for a critical role of the *temporal* organization of neural processing, providing evidence for the temporal hypothesis of compensation in age (Dew et al., 2011; Martins et al., 2015). Hence, in future studies, our fMRI-based results should be cross-validated by techniques with a higher temporal resolution, such as EEG techniques.

### Further Limitations and Outlook

From a methodological perspective, it might be criticized first that we did not analyze the transient and sustained processing dynamics within the same task. A more suitable technical way to compare transient versus sustained components of brain activation would be to estimate parameters for both trial-related (transient) and block-related (sustained) effects in the task simultaneously but to enter them as independent regressors within the same analysis design, such as in the mixed block/event-related design. The mixed block/event-related analysis approach has been validated well through both empirical and simulation studies (Braver et al., 2003, 2009; Visscher et al., 2003; Dennis et al., 2007). However, this analysis forfeits a lot of statistical power due to the loss of degrees of freedom by the large number of sustained and transient regressors that have to be modeled, especially in case of an intervention study. We would have needed to considerably expand the amount of experimental trials per task in our study to obtain an adequate experimental design that can be analyzed with such mixed analysis procedures (Visscher et al., 2003). This was, however, not feasible in the present case for reasons of testing logistics. Future studies may consider splitting their tasks measurement into several sessions, or alternatively, could focus on the measurement of only one untrained additional task, which would allow a larger number of trials to be included per task per session. Hence, this would probably provide more robust effect sizes for any obtainable changes.

A second caveat might be that we captured the sustained effects by relatively lengthy scanning runs. As tested by Visscher et al. (2003), one advantage of such a long recording time for the sustained response is that extending the length of a scanning run by increasing the number of task frames in the modeled sustained response seems to decrease the covariance of parameters (i.e., the amount that the estimate of one parameter is affected by the estimate of another parameter; see also Petersen and Dubis, 2012). However, the upper bound of this limit has not been tested sufficiently and task efficiency, as influenced by factors like fatigue effects and scanner drift, has to be taken into account.

A third concern is that we relied exclusively on an ROI-based approach. ROI-based approaches have the advantage that they have more statistical power than whole-brain analyses because the number of statistical comparisons is greatly reduced (Poldrack et al., 2011), which was especially relevant in the present study facing the highly variable data of older adults. In the present study, we were specifically interested in effects within the ROI-masks as defined in the section “Materials and Methods,” while not in the whole-brain mask. However, such an approach may also prevent one from discovering other regions outside the ROI-masks that could be involved in the mechanisms of interest. Extensions of the present results could combine whole-brain and ROI-based approaches to obtain a wider picture, if they made modifications to attempt to increase power in the design.

Fourth, the scope of neural plasticity cannot be phenotyped exhaustively by considering only changes in brain functioning in untrained tasks but may also require the investigation of structural adaptations of brain tissue, such as gray and white matter. Recent reviews on the cognitive neuroscience of aging attested the great extent of neuroanatomical changes, with an approximately linear macro-level decline in brain weight and volume of 2% per decade, and an additional expansion of cerebral ventricles and sulci (Reuter-Lorenz and Mikels, 2006). These macro-structural alterations become apparent in changes at the micro level, such as shrinkage of neuronal cell bodies, loss of synaptic density, debranching of dendritic arbors, depletion of dopamine receptors, and especially damage to connecting white matter fibers. Although we know about the limited potential to plasticity in later stages of the lifespan, the extent to which executive-control training can boost such structural changes in older adults’ brain morphometry has not been thoroughly examined yet.

Fifth, beyond the reliable average benefit, there is accumulating evidence for large inter-individual heterogeneity in initial resources as well as in practice-induced changes (Karbach and Kray, 2016). In particular, older adults start with very different ability profiles because their learning histories have diverged over a lifetime. Hence, future research should analyze the scope of practice-induced plasticity in old age as a function of inter-individual differences in baseline capacities and training gains.

Given these analysis limitations, our data should be interpreted only with caution. However, the present study provides important new insights into the neural mechanisms underlying training-induced improvement in executive-control functioning, albeit exploratory by nature. Hence, to uncover the latent potential of the aging brain and scope of neural plasticity from cognitive interventions in old age, interactions between spatial and especially temporal dynamics of brain activation should be considered.

## Ethics Statement

This study was carried out in accordance with the recommendations of the local ethics committee at Saarland University. The protocol was approved by the local ethics committee at Saarland University.

## Author Contributions

SD carried out the statistical analyses and interpretations and contributed the most to the writing of the manuscript. CS, CW, and MW supported writing of the methods and results sections. JK and HZ were the principal investigator. JK, CW, CS, MW, and HZ provided substantial support in proofreading and revising the manuscript and gave the final approval of the version to be published. Intellectual content was collectively revised by all authors. All authors read and approved the final manuscript.

## Conflict of Interest

The authors declare that the research was conducted in the absence of any commercial or financial relationships that could be construed as a potential conflict of interest.

## Footnotes

1 Stimuli were mainly retrieved from the databases of Snodgrass and Vanderwart (1980) and Rossion and Pourtois (2004).

2 Taken from the database of Minear and Shah (2008).

3 Taken from the databases of Snodgrass and Vanderwart (1980) and of Rossion and Pourtois (2004).

4 176 grayscale landscape photos were kindly provided by the Gazzaley Lab from their database.

5 248 grayscale photos of neutral female and male (124 each) faces from a large age range were kindly provided by the Gazzaley Lab from their database.

6 It should be noted that the gender of catch trials was also counterbalanced across participants.

7 http://www.fil.ion.ucl.ac.uk/spm/

8 http://dbm.neuro.uni-jena.de/cat12/
