# Supplementary material for: Spatio-Temporal Neural Changes After Task-Switching Training in Old Age
Source: Front Aging Neurosci. 2019 Oct 15;11:267. doi: 10.3389/fnagi.2019.00267 (PMC6803514; doi:10.3389/fnagi.2019.00267)
Supplement: Supplementary file 1 [file Table_1.DOCX]

**Supplementary Material**

**SI1.** Description of the customized longitudinal fMRI data preprocessing pipeline

For each run, the first four functional images of each subject were discarded to allow for equilibrium of longitudinal magnetization. We manually set the origin of each subject’s structural and functional images to correspond approximately to the AC point. Functional images were temporally aligned to the middle slice of the first scanning run per session, using SPM12’s Fourier phase shift interpolation. To correct for head motion, slice-time corrected functional images were realigned to the mid-point average functional image from both sessions by a least squares approach and a 6 parameter (rigid body) spatial transformation as implemented in SPM12, using a 5^th^ degree b-spline interpolation. For the co-registration of functional and structural images, we first registered the posttest structural images to the pretest structural images using the longitudinal rigid registration pipeline of CAT12 to obtain a mid-point average output image from both sessions. In contrast to the default preprocessing pipeline of SPM12, the CAT12 longitudinal processing pipeline is optimized for the detection of subtle changes over shorter time ranges, such as brain plasticity or training effects after a few weeks, and therefore seemed especially suited for the present study. Slice-time-corrected and realigned functional images from both sessions were then co-registered to the mid-point average structural image. The mid-point average structural image was skull-stripped, bias-field and intensity-corrected, and then segmented into grey and white matter, and cerebrospinal fluid. To allow for inter-individual comparisons, spatial normalization parameters for mapping each subject’s anatomy to a common template in MNI stereotactic space were derived by iteratively registering the segmented mid-point average image via a high-dimensional fast diffeomorphic registration algorithm (DARTEL; Ashburner & Friston, 2011) to the CAT12 default template (IXI555_MNI152, 1.5 x 1.5 x 1.5 mm^3^). The latter template is generated based on the T1-weighted images of nearly 600 healthy adults of ages ranging from 19.98 to 86.32 years (averaged across relatively equal numbers of older and younger adult brains), thus providing a good basis for age-unbiased registration. Non-linear forward deformation fields were estimated, defined as displacement vectors at each voxel representing the transformation needed to map a voxel of the template to its corresponding position in the subject brain. These forward deformations were applied to all functional images of all subjects, using a 7^th^ degree b-spline interpolation. All normalized functional series were then re-sampled to a resolution of 2 x 2 x 2 mm^3^. As a final step, data were spatially smoothed via the SPM standard module with an 8-mm FWHM (full width at half maximum) Gaussian kernel applied to ameliorate differences in inter-subject localization. For model estimation, we modified the SPM default masking procedure that masks out voxels failing to exceed an arbitrary implicit masking threshold of 0.8, which can lead to the non-analysis of, for example, frontopolar regions (see <http://akiraoconnor.org/2010/04/07/masking-in-spm/>) that were, however, critical in the present study. As recommended, we lowered the masking threshold to 0.2 and set instead an explicit inclusive mask in the model specification stage based on a custom average whole-brain image of our study sample. To create this explicit mask, we took the individual skull-stripped, bias-field and intensity-corrected structural mid-point average images of all our subjects, and calculated a mean image by applying nearest-neighbor interpolation in SPM’s ImCalc functionality. This binarized mean image was resampled to the voxel size of the pre-processed functional data (i.e., 2 x 2 x 2 mm^3^).

**SI2.** Description of the computation procedure of literature-based probabilistic ROIs

First, we created anatomical ROIs within the PFC-PPC that have been shown to be primarily sensitive to transient modulations, including the mid-ventrolateral and dorsolateral PFC, the inferior frontal junction, as well as the posterior parietal cortex, subdivided into its superior and inferior parts. Second, we created anatomical ROIs within the PFC-BG circuitry that have been shown to be primarily sensitive to sustained modulations, including the frontal pole, the anterior part of the cingulate cortex, and, sub-cortically, the basal ganglia. Anatomical ROIs were created based on the SPM Anatomy toolbox v2.2b (Eickhoff et al., 2005). Next, spatial coordinates for these ROIs were taken from meta-analyses using comparable task designs to our study (refer to the following table and reference list). If these coordinates were given in TAL (Talairach) instead of MNI coordinates, they were first transformed into MNI coordinates. Based on both anatomical and functional information, we created the ROI masks in a three-step process as described in Schubert et al. (2008):

(1) The probability that a voxel at a given position within an anatomical ROI showed neural activation regarding the corresponding literature was estimated by calculating a 3D normal (Gaussian) distribution G(x, y, z) as follows (Turkeltaub et al., 2002):

where C is the covariance matrix for all coordinate triples x, y, z from the underlying literature and x, y, z are the mean values of the x, y, and z coordinates, respectively (Nielsen et al., 2002).

(2) The outer limits of the finally used ROI were defined by (a) the outer limits of the anatomical ROI and (b) a threshold of 2 SDs of the resulting 3D distribution.

(3) A binary mask including all voxels spatially within these boundaries was formed (with a default resolution of 2 x 2 x 2 mm^3^).

For spatially extended anatomical ROIs that probably contain different functional sub-regions, this procedure leads to a spatial reduction to design relevant coordinates within these ROIs.

For further analyses, each ROI was intersected with the customized whole-brain structural mask from our study sample (see SI1).

*Overview of all literature-based probabilistic regions of interest. Red area reflects atlas-based anatomical borders; blue dots reflect coordinates retrieved from the literature; green area reflects the computed probabilistic region of interest.*

**
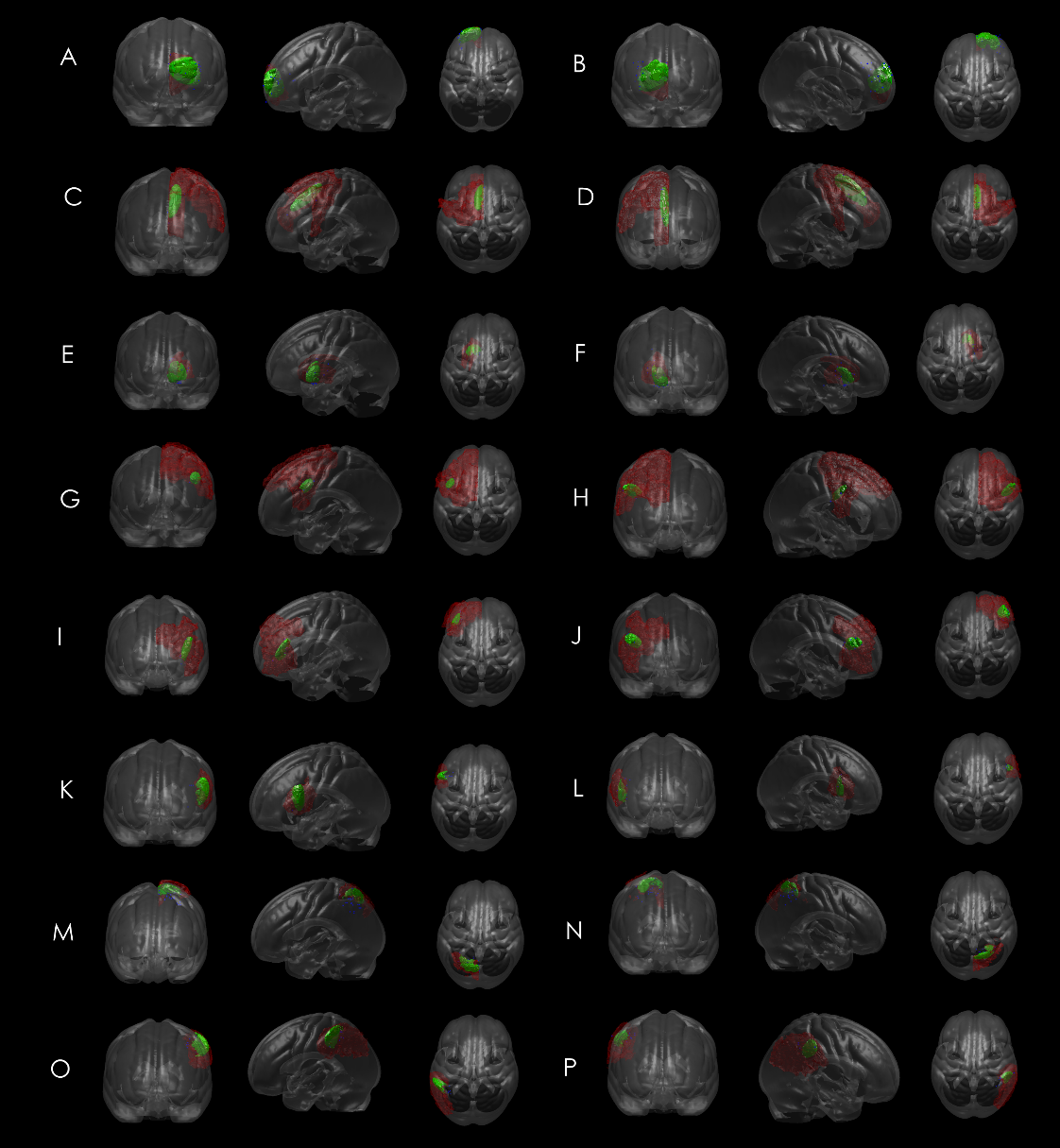
**

**A.** = Frontal pole (FP) left. **B.** = Frontal pole (FP) right. **C.** =Anterior cingulate cortex (ACC) left. **D.** = Anterior cingulate cortex (ACC) right. **E.** = Basal ganglia (BG) left. **F.** = Basal ganglia (BG) right. **G.** = Inferior frontal junction (IFJ) left. **H.** = Inferior frontal junction (IFJ) right. **I.** = Dorsolateral prefrontal cortex (dlPFC) left. **J.** = dorsolateral prefrontal cortex (dlPFC) right. **K.** = Ventrolateral prefrontal cortex (vlPFC) left. **L.** = Ventrolateral prefrontal cortex (vlPFC) right. **M.** = Superior parietal lobule (SPL) left. **N.** = Superior parietal lobule (SPL) right. **O.** = Inferior parietal lobule (IPL) left. **P.** = Inferior parietal lobule (IPL) right.

*Overview of all functional coordinates and of meta-analyses from which they were retrieved for the computation of literature-based probabilistic regions of interest used to mask the neuroimaging data (from anterior to posterior ROIs).*

| ROI |  | Coordinates | | | | Meta-analysis |
| --- | --- | --- | --- | --- | --- | --- |
|  |  | x | y | z | MNI/ TAL |  |
| FP | L | -33 | 54 | 3 | MNI | Gilbert et al. (2006) |
|  |  | -2 | 63 | 27 | MNI | Gilbert et al. (2006) |
|  |  | -34 | 50 | 9 | MNI | Gilbert et al. (2006) |
|  |  | -20 | 70 | 4 | MNI | Gilbert et al. (2006) |
|  |  | 0 | 64 | 8 | MNI | Gilbert et al. (2006) |
|  |  | -36 | 60 | -4 | MNI | Gilbert et al. (2006) |
|  |  | 0 | 64 | 16 | MNI | Gilbert et al. (2006) |
|  |  | -36 | 53 | 12 | MNI | Gilbert et al. (2006) |
|  |  | 0 | 54 | 3 | MNI | Gilbert et al. (2006) |
|  |  | -32 | 55 | 13 | MNI | Gilbert et al. (2006) |
|  |  | -32 | 61 | 16 | MNI | Gilbert et al. (2006) |
|  |  | -32 | 50 | 8 | TAL | Kim, Cilles, Johnson, and Gold (2012) |
|  |  | -34 | 50 | 22 | TAL | Kim et al. (2012) |
|  |  | -10 | 54 | 18 | TAL | Kim et al. (2012) |
|  |  | -12 | 48 | 4 | TAL | Kim et al. (2012) |
|  |  | -38 | 52 | 10 | MNI | Nee et al. (2013) |
|  |  | -38 | 52 | 8 | MNI | Nee et al. (2013), Supplemental Table II |
|  |  | -36 | 38 | 20 | MNI | Nee, Wager, and Jonides (2007) |
|  |  | -36 | 44 | 20 | TAL | Owen, McMillan, Laird, and Bullmore (2005) |
|  |  | -32 | 42 | 10 | TAL | Owen et al. (2005) |
|  |  | -28 | 62 | -4 | TAL | Owen et al. (2005) |
|  |  | -32 | 54 | 6 | MNI | Wager, Jonides, and Reading (2004) |
|  |  | -32 | 34 | 11 | MNI | Wager and Smith (2003) |
|  |  | -33 | 32 | 12 | MNI | Wager and Smith (2003) |
| FP | R | 37 | 49 | 19 | MNI | Gilbert et al. (2006) |
|  |  | 40 | 51 | 3 | MNI | Gilbert et al. (2006) |
|  |  | 30 | 65 | 27 | MNI | Gilbert et al. (2006) |
|  |  | 34 | 64 | -4 | MNI | Gilbert et al. (2006) |
|  |  | 0 | 64 | 8 | MNI | Gilbert et al. (2006) |
|  |  | 8 | 56 | 28 | MNI | Gilbert et al. (2006) |
|  |  | 6 | 53 | 16 | MNI | Gilbert et al. (2006) |
|  |  | 0 | 64 | 26 | MNI | Gilbert et al. (2006) |
|  |  | 34 | 60 | 4 | MNI | Gilbert et al. (2006) |
|  |  | 0 | 54 | 3 | MNI | Gilbert et al. (2006) |
|  |  | 38 | 59 | 10 | MNI | Gilbert et al. (2006) |
|  |  | 30 | 75 | 12 | MNI | Gilbert et al. (2006) |
|  |  | 36 | 66 | 21 | MNI | Gilbert et al. (2006) |
|  |  | 9 | 42 | -6 | MNI | Gilbert et al. (2006) |
|  |  | 15 | 63 | 21 | MNI | Gilbert et al. (2006) |
|  |  | 26 | 63 | 10 | MNI | Gilbert et al. (2006) |
|  |  | 38 | 47 | 11 | MNI | Gilbert et al. (2006) |
|  |  | 25 | 55 | 4 | MNI | Gilbert et al. (2006) |
|  |  | 22 | 49 | 10 | MNI | Gilbert et al. (2006) |
|  |  | 33 | 49 | 28 | MNI | Gilbert et al. (2006) |
|  |  | 32 | 48 | 16 | TAL | Kim et al. (2012) |
|  |  | 42 | 46 | 26 | MNI | Nee et al. (2013) |
|  |  | 32 | 38 | 22 | MNI | Nee et al. (2007) |
|  |  | 34 | 54 | -8 | MNI | Wager et al. (2004) |
|  |  | 36 | 36 | 28 | MNI | Wager and Smith (2003) |
| ACC | L | -8 | 20 | 42 | TAL | Derrfuss, Brass, Neumann, and von Cramon (2005) |
|  |  | -2 | 36 | 26 | TAL | Derfuss et al. (2005) |
|  |  | -8 | 10 | 48 | TAL | Kim et al. (2012) |
|  |  | 0 | 18 | 50 | MNI | Nee et al. (2013) |
|  |  | -2 | 24 | 38 | MNI | Nee et al. (2013) |
|  |  | -2 | 26 | 34 | MNI | Nee et al. (2013), Supplemental Table II |
|  |  | -2 | 4 | 62 | MNI | Nee et al. (2013), Supplemental Table II |
|  |  | 0 | 8 | 54 | MNI | Nee et al. (2007) |
|  |  | 0 | 20 | 40 | MNI | Nee et al. (2007) |
|  |  | -2 | 26 | 42 | MNI | Nee et al. (2007) |
|  |  | -8 | 8 | 52 | MNI | Nee et al. (2007) |
|  |  | -6 | 4 | 40 | MNI | Nee et al. (2007) |
|  |  | -2 | 12 | 42 | TAL | Owen et al. (2005) |
|  |  | 0 | 26 | 36 | TAL | Owen et al. (2005) |
|  |  | -2 | 12 | 42 | TAL | Owen et al. (2005) |
|  |  | -1 | 5 | 52 | MNI | Wager and Smith (2003) |
|  |  | 0 | 11 | 49 | MNI | Wager and Smith (2003) |
|  |  | -18 | 8 | 52 | MNI | Wager et al. (2004) |
|  |  | -10 | 9 | 50 | MNI | Wager et al. (2004) |
| ACC | R | 2 | 14 | 42 | TAL | Derfuss et al. (2005) |
|  |  | 4 | 8 | 48 | TAL | Derfuss et al. (2005) |
|  |  | 4 | 28 | 42 | TAL | Derfuss et al. (2005) |
|  |  | 4 | 26 | 41 | TAL | Derrfuss, Brass, and Von Cramon (2004) |
|  |  | 8 | 8 | 68 | MNI | Nee et al. (2013) |
|  |  | 0 | 18 | 50 | MNI | Nee et al. (2013) |
|  |  | 4 | 16 | 52 | MNI | Nee et al. (2013), Supplemental Table II |
|  |  | 6 | 8 | 68 | MNI | Nee et al. (2013), Supplemental Table II |
|  |  | 0 | 8 | 54 | MNI | Nee et al. (2007) |
|  |  | 0 | 20 | 40 | MNI | Nee et al. (2007) |
|  |  | 2 | 16 | 46 | MNI | Nee et al. (2007) |
|  |  | 0 | 4 | 36 | MNI | Nee et al. (2007) |
|  |  | 10 | 4 | 50 | MNI | Nee et al. (2007) |
|  |  | 0 | 26 | 36 | TAL | Owen et al. (2005) |
|  |  | 0 | 26 | 36 | TAL | Owen et al. (2005) |
|  |  | 0 | 11 | 49 | MNI | Wager and Smith (2003) |
|  |  | 10 | 15 | 43 | MNI | Wager et al. (2004) |
| BG | L | -12 | 12 | -6 | MNI | Bartra, McGuire, and Kable (2013) |
|  |  | -12 | 4 | 2 | MNI | Bartra et al. (2013) |
|  |  | -14 | 10 | -6 | MNI | Bartra et al. (2013) |
|  |  | -16 | 4 | -4 | MNI | Bartra et al. (2013) |
|  |  | -6 | 8 | -4 | MNI | Bartra et al. (2013) |
|  |  | -8 | 8 | -6 | MNI | Bartra et al. (2013) |
|  |  | -4 | 10 | -2 | MNI | Bartra et al. (2013) |
|  |  | -6 | 10 | -6 | MNI | Bartra et al. (2013) |
|  |  | -14 | 2 | -12 | MNI | Bartra et al. (2013) |
|  |  | -10 | 10 | -2 | MNI | Diekhof, Kaps, Falkai, and Gruber (2012) |
|  |  | -10 | 12 | -6 | MNI | Diekhof et al. (2012) |
|  |  | -10 | 6 | 2 | MNI | Diekhof et al. (2012) |
|  |  | -10 | 8 | -12 | MNI | Diekhof et al. (2012) |
|  |  | -14 | 14 | 0 | MNI | Diekhof et al. (2012) |
|  |  | -16 | 10 | -14 | MNI | Diekhof et al. (2012) |
|  |  | -8 | 16 | -8 | MNI | Diekhof et al. (2012) |
|  |  | -18 | 6 | -12 | MNI | Garrison, Erdeniz, and Done (2013) |
|  |  | -12 | 6 | 6 | MNI | Garrison et al. (2013) |
|  |  | -22 | 16 | 4 | MNI | Garrison et al. (2013) |
|  |  | -10 | 6 | 8 | MNI | Garrison et al. (2013) |
|  |  | -24 | 6 | 6 | MNI | Garrison et al. (2013) |
|  |  | -22 | 16 | 4 | MNI | Garrison et al. (2013) |
|  |  | -18 | 2 | 8 | MNI | Garrison et al. (2013) |
|  |  | -20 | 8 | -14 | MNI | Garrison et al. (2013) |
|  |  | -10 | 8 | 2 | MNI | Garrison et al. (2013) |
|  |  | -18 | 8 | -12 | MNI | Garrison et al. (2013) |
|  |  | -8 | 4 | 18 | MNI | Garrison et al. (2013) |
|  |  | -18 | 2 | 8 | MNI | Garrison et al. (2013) |
|  |  | -10 | 4 | 10 | MNI | Garrison et al. (2013) |
|  |  | -28 | -6 | 2 | MNI | Garrison et al. (2013) |
|  |  | -5 | 1 | 16 | MNI | Garrison et al. (2013) |
|  |  | -10 | 3 | 18 | MNI | Garrison et al. (2013) |
|  |  | -30 | 6 | 6 | MNI | Groenewold, Opmeer, de Jonge, Aleman, and Costafreda (2013) |
|  |  | -20 | -2 | 14 | TAL | Jamadar, Fielding, and Egan (2013) |
|  |  | -12 | 10 | -4 | MNI | Kaps (2012) |
|  |  | -10 | 12 | -6 | MNI | Kaps (2012) |
|  |  | -16 | 12 | -10 | MNI | Liu, Hairston, Schrier, and Fan (2011) |
|  |  | -12 | 10 | -6 | MNI | Liu et al. (2012) |
|  |  | -10 | 8 | -4 | MNI | Liu et al. (2012) |
|  |  | -16 | 4 | -14 | MNI | Liu et al. (2012) |
|  |  | -26 | 6 | -8 | MNI | Liu et al. (2012) |
|  |  | -10 | 10 | -4 | MNI | Liu et al. (2012) |
|  |  | -24 | 4 | 6 | MNI | Liu et al. (2012) |
|  |  | -18 | 8 | -14 | MNI | Liu et al. (2012) |
|  |  | -8 | 14 | 2 | MNI | Liu et al. (2012) |
|  |  | -8 | 8 | -4 | MNI | Liu et al. (2012) |
|  |  | -16 | 4 | -10 | MNI | Liu et al. (2012) |
|  |  | -8 | 6 | -6 | MNI | Liu et al. (2012) |
|  |  | -16 | 8 | -8 | MNI | Liu et al. (2012) |
|  |  | -18 | 4 | -14 | MNI | Liu et al. (2012) |
|  |  | -18 | 4 | -10 | MNI | Liu et al. (2012) |
|  |  | -16 | 4 | -10 | MNI | Liu et al. (2012) |
|  |  | -16 | 8 | -10 | MNI | Liu et al. (2012) |
|  |  | -10 | 8 | -4 | MNI | Liu et al. (2012) |
|  |  | -20 | 6 | -12 | MNI | Liu et al. (2012) |
|  |  | -6 | 4 | -10 | MNI | Liu et al. (2012) |
|  |  | -14 | -11 | 3 | MNI | Wager and Smith (2003) |
|  |  | -5 | -12 | 9 | MNI | Wager and Smith (2003) |
| BG | R | 12 | 10 | -6 | MNI | Bartra et al. (2013) |
|  |  | 12 | 10 | -2 | MNI | Bartra et al. (2013) |
|  |  | 14 | 12 | -10 | MNI | Bartra et al. (2013) |
|  |  | 12 | 6 | -8 | MNI | Bartra et al. (2013) |
|  |  | 12 | 6 | 4 | MNI | Bartra et al. (2013) |
|  |  | 18 | 6 | -6 | MNI | Bartra et al. (2013) |
|  |  | 6 | 10 | -8 | MNI | Bartra et al. (2013) |
|  |  | 12 | 10 | -6 | MNI | Bartra et al. (2013) |
|  |  | 8 | 10 | -6 | MNI | Bartra et al. (2013) |
|  |  | 14 | 14 | -6 | MNI | Bartra et al. (2013) |
|  |  | 10 | 10 | 2 | MNI | Bartra et al. (2013) |
|  |  | 10 | 12 | -6 | MNI | Bartra et al. (2013) |
|  |  | 20 | 14 | -8 | MNI | Bartra et al. (2013) |
|  |  | 12 | 14 | -4 | MNI | Diekhof et al. (2012) |
|  |  | 20 | 8 | 10 | MNI | Diekhof et al. (2012) |
|  |  | 16 | 12 | -12 | MNI | Diekhof et al. (2012) |
|  |  | 18 | 22 | 2 | MNI | Diekhof et al. (2012) |
|  |  | 12 | 12 | -4 | MNI | Diekhof et al. (2012) |
|  |  | 10 | 16 | -8 | MNI | Diekhof et al. (2012) |
|  |  | 12 | 12 | -8 | MNI | Diekhof et al. (2012) |
|  |  | 14 | 12 | -12 | MNI | Diekhof et al. (2012) |
|  |  | 20 | 12 | -12 | MNI | Diekhof et al. (2012) |
|  |  | 8 | 20 | -6 | MNI | Diekhof et al. (2012) |
|  |  | 10 | 16 | -12 | MNI | Diekhof et al. (2012) |
|  |  | 28 | -4 | 2 | MNI | Diekhof et al. (2012) |
|  |  | 16 | 14 | -10 | MNI | Diekhof et al. (2012) |
|  |  | 10 | 6 | -2 | MNI | Garrison et al. (2013) |
|  |  | 22 | 4 | 12 | MNI | Garrison et al. (2013) |
|  |  | 12 | 8 | -4 | MNI | Garrison et al. (2013) |
|  |  | 10 | 8 | 2 | MNI | Garrison et al. (2013) |
|  |  | 8 | 10 | -14 | MNI | Garrison et al. (2013) |
|  |  | 10 | 14 | -10 | MNI | Garrison et al. (2013) |
|  |  | 10 | 6 | -2 | MNI | Garrison et al. (2013) |
|  |  | 22 | 2 | 6 | MNI | Garrison et al. (2013) |
|  |  | 28 | -4 | 8 | MNI | Groenewold et al. (2013) |
|  |  | 18 | -4 | 16 | TAL | Jamadar et al. (2013) |
|  |  | 12 | 12 | -4 | MNI | Kaps (2012) |
|  |  | 4 | -18 | -14 | MNI | Kaps (2012) |
|  |  | 14 | 14 | -10 | MNI | Kaps (2012) |
|  |  | 12 | 10 | -6 | MNI | Liu et al. (2012) |
|  |  | 12 | 10 | -8 | MNI | Liu et al. (2012) |
|  |  | 10 | 6 | 4 | MNI | Liu et al. (2012) |
|  |  | 12 | 10 | -4 | MNI | Liu et al. (2012) |
|  |  | 24 | 4 | 0 | MNI | Liu et al. (2012) |
|  |  | 10 | 10 | -10 | MNI | Liu et al. (2012) |
|  |  | 20 | 4 | 18 | MNI | Liu et al. (2012) |
|  |  | 12 | 8 | -4 | MNI | Liu et al. (2012) |
|  |  | 14 | 10 | -12 | MNI | Liu et al. (2012) |
|  |  | 8 | 20 | 2 | MNI | Liu et al. (2012) |
|  |  | 12 | 8 | -10 | MNI | Liu et al. (2012) |
|  |  | 14 | 10 | -10 | MNI | Liu et al. (2012) |
|  |  | 12 | 2 | -10 | MNI | Liu et al. (2012) |
|  |  | 14 | 8 | -14 | MNI | Liu et al. (2012) |
|  |  | 12 | 2 | -4 | MNI | Liu et al. (2012) |
|  |  | 12 | 12 | -6 | MNI | Liu et al. (2012) |
|  |  | 14 | 8 | -12 | MNI | Liu et al. (2012) |
|  |  | 12 | 2 | -10 | MNI | Liu et al. (2012) |
|  |  | 8 | 12 | -8 | MNI | Liu et al. (2012) |
|  |  | 20 | -14 | 30 | TAL | Murty, Ritchey, Adcock, and LaBar (2010) |
| DLPFC | L | -41 | 22 | 29 | TAL | Derrfuss et al. (2004) |
|  |  | -42 | 23 | 27 | TAL | Derfuss et al. (2004) |
|  |  | -34 | 30 | 18 | TAL | Kim et al. (2012) |
|  |  | -40 | 26 | 24 | TAL | Owen et al. (2005) |
|  |  | -32 | 42 | 10 | TAL | Owen et al. (2005) |
|  |  | -44 | 28 | 28 | MNI | Nee et al. (2013) |
|  |  | -40 | 26 | 30 | MNI | Nee et al. (2007) |
|  |  | -36 | 38 | 20 | MNI | Nee et al. (2007) |
|  |  | -40 | 32 | 34 | MNI | Nee et al. (2007) |
|  |  | -32 | 34 | 11 | MNI | Wager and Smith (2003) |
|  |  | -33 | 32 | 12 | MNI | Wager and Smith (2003) |
| DLPFC | R | 46 | 28 | 24 | TAL | Derrfuss et al. (2005) |
|  |  | 40 | 32 | 24 | MNI | Nee et al. (2013) |
|  |  | 42 | 46 | 26 | MNI | Nee et al. (2013) |
|  |  | 50 | 32 | 34 | MNI | Nee et al. (2013) |
|  |  | 38 | 34 | 26 | MNI | Nee et al. (2013), Supplemental Table I |
|  |  | 50 | 38 | 22 | MNI | Nee et al. (2013), Supplemental Table II |
|  |  | 50 | 36 | 30 | MNI | Nee et al. (2013), Supplemental Table II |
|  |  | 42 | 22 | 24 | MNI | Nee et al. (2007) |
|  |  | 42 | 24 | 28 | MNI | Nee et al. (2007) |
|  |  | 40 | 32 | 30 | TAL | Owen et al. (2005) |
|  |  | 42 | 30 | 24 | TAL | Owen et al. (2005) |
|  |  | 36 | 36 | 28 | MNI | Wager and Smith (2003) |
|  |  | 42 | 38 | 28 | MNI | Wager et al. (2004) |
| Mid-VLPFC | L | -48 | 14 | 18 | TAL | Derfuss et al. (2005) |
|  |  | -48 | 10 | 4 | TAL | Kim et al. (2012) |
|  |  | -52 | 12 | 22 | MNI | Nee et al. (2013) |
|  |  | -50 | 20 | 28 | MNI | Nee et al. (2013) |
|  |  | -48 | 22 | 26 | MNI | Nee et al. (2013), Supplemental Table I |
|  |  | -40 | 16 | 28 | MNI | Nee et al. (2013), Supplemental Table I |
|  |  | -54 | 14 | 22 | MNI | Nee et al. (2013), Supplemental Table II |
|  |  | -50 | 20 | 28 | MNI | Nee et al. (2013), Supplemental Table II |
|  |  | -38 | 16 | 28 | MNI | Nee et al. (2013), Supplemental Table II |
|  |  | -36 | 22 | 2 | MNI | Nee et al. (2013), Supplemental Table II |
|  |  | -42 | 16 | 28 | MNI | Nee et al. (2007) |
|  |  | -44 | 18 | 22 | TAL | Owen et al. (2005) |
| Mid-VLPFC | R | 42 | 11 | 7 | TAL | Derfuss et al. (2004) |
|  |  | 44 | 18 | 4 | MNI | Nee et el. (2013) |
|  |  | 56 | 12 | 14 | MNI | Nee et al. (2013), Supplemental Table II |
|  |  | 44 | 14 | 8 | MNI | Nee et al. (2007) |
|  |  | 44 | 20 | 22 | MNI | Nee et al. (2007) |
|  |  | 40 | 14 | 28 | MNI | Nee et al. (2007) |
|  |  | 46 | 16 | 30 | MNI | Nee et al. (2007) |
| IFJ | L | -40 | 4 | 30 | TAL | Derfuss et al. (2004) |
|  |  | -40 | 4 | 32 | TAL | Derfuss et al. (2005) |
|  |  | -38 | 2 | 32 | TAL | Derfuss et al. (2004) |
|  |  | -47 | 5 | 30 | TAL | Derfuss et al. (2004) |
|  |  | -36 | 10 | 30 | TAL | Kim et al. (2012) |
|  |  | -40 | 4 | 36 | TAL | Kim et al. (2012) |
|  |  | -42 | 0 | 30 | TAL | Kim et al. (2012) |
|  |  | -46 | -2 | 40 | MNI | Nee et al. (2013), Supplemental Table II |
|  |  | -40 | 4 | 38 | MNI | Nee et al. (2007) |
|  |  | -44 | 10 | 38 | MNI | Nee et al. (2007) |
|  |  | -40 | 10 | 38 | MNI | Nee et al. (2007) |
|  |  | -44 | -2 | 38 | TAL | Owen et al. (2005) |
|  |  | -44 | 4 | 32 | TAL | Owen et al. (2005) |
|  |  | -43 | 2 | 37 | MNI | Wager and Smith (2003) |
|  |  | -38 | 10 | 36 | MNI | Wager et al. (2004) |
| IFJ | R | 44 | 10 | 34 | TAL | Derfuss et al. (2005) |
|  |  | 47 | 8 | 30 | TAL | Derfuss et al. (2004) |
|  |  | 46 | -2 | 28 | TAL | Kim et al. (2012) |
|  |  | 48 | 4 | 38 | MNI | Nee et al. (2013) |
|  |  | 40 | 6 | 38 | MNI | Nee et al. (2013) |
|  |  | 48 | 6 | 38 | MNI | Nee et al. (2013), Supplemental Table II |
|  |  | 48 | 4 | 38 | MNI | Nee et al. (2013), Supplemental Table II |
|  |  | 38 | -2 | 33 | MNI | Wager and Smith (2003) |
|  |  | 45 | 1 | 29 | MNI | Wager and Smith (2003) |
| SPL | L | -4 | -61 | 47 | TAL | Derfuss et al. (2004) |
|  |  | -26 | -66 | 32 | TAL | Kim et al. (2012) |
|  |  | -2 | -76 | 40 | TAL | Kim et al. (2012) |
|  |  | -10 | -54 | 48 | MNI | Kim et al. (2012) |
|  |  | -4 | -68 | 48 | TAL | Kim et al. (2012) |
|  |  | -20 | -66 | 58 | TAL | Kim et al. (2012) |
|  |  | -22 | -74 | 44 | TAL | Kim et al. (2012) |
|  |  | -10 | -70 | 62 | MNI | Nee et al. (2013) |
|  |  | -12 | -72 | 46 | MNI | Nee et al. (2013) |
|  |  | -18 | -74 | 42 | MNI | Nee et al. (2013) |
|  |  | -22 | -64 | 54 | MNI | Nee et al. (2013) |
|  |  | -28 | -66 | 36 | MNI | Nee et al. (2013) |
|  |  | -32 | -48 | 60 | MNI | Nee et al. (2013), Supplemental Table I and II |
|  |  | -8 | -66 | 62 | MNI | Nee et al. (2013), Supplemental Table II |
|  |  | -14 | -64 | 54 | MNI | Nee et al. (2013), Supplemental Table II |
|  |  | -10 | -56 | 48 | MNI | Nee et al. (2013), Supplemental Table II |
|  |  | -12 | -72 | 62 | MNI | Nee et al. (2013), Supplemental Table II |
|  |  | -18 | -58 | 54 | MNI | Nee et al. (2013), Supplemental Table II |
|  |  | -24 | -64 | 56 | MNI | Nee et al. (2013), Supplemental Table II |
|  |  | -22 | -62 | 48 | MNI | Nee et al. (2013), Supplemental Table II |
|  |  | -34 | -62 | 54 | MNI | Nee et al. (2013), Supplemental Table II |
|  |  | -18 | -72 | 42 | MNI | Nee et al. (2007) |
|  |  | -16 | -62 | 48 | MNI | Nee et al. (2007) |
|  |  | -22 | -64 | 46 | MNI | Nee et al. (2007) |
|  |  | -30 | -54 | 40 | TAL | Owen et al. (2005) |
|  |  | -31 | -61 | 38 | MNI | Wager and Smith (2003) |
|  |  | -12 | -70 | 46 | MNI | Wager and Smith (2003) |
| SPL | R | 30 | -56 | 52 | TAL | Kim et al. (2012) |
|  |  | 16 | -62 | 50 | MNI | Kim et al. (2012) |
|  |  | 4 | -56 | 54 | MNI | Nee et al. (2013), Supplemental TableI and II |
|  |  | 8 | -60 | 46 | MNI | Nee et al. (2013), Supplemental Table II |
|  |  | 10 | -60 | 68 | MNI | Nee et al. (2013) |
|  |  | 12 | -66 | 60 | MNI | Nee et al. (2013), Supplemental Table II |
|  |  | 14 | -68 | 62 | MNI | Nee et al. (2013), Supplemental Table II |
|  |  | 14 | -66 | 60 | MNI | Nee et al. (2013), Supplemental Table II |
|  |  | 14 | -56 | 50 | MNI | Nee et al. (2013), Supplemental Table II |
|  |  | 16 | -60 | 54 | MNI | Nee et al. (2013) |
|  |  | 18 | -72 | 50 | MNI | Nee et al. (2013) |
|  |  | 18 | -60 | 58 | MNI | Nee et al. (2013), Supplemental Table II |
|  |  | 26 | -66 | 38 | MNI | Nee et al. (2013) |
|  |  | 28 | -56 | 60 | MNI | Nee et al. (2013), Supplemental Table I |
|  |  | 22 | -66 | 58 | MNI | Nee et al. (2013), Supplemental Table II |
|  |  | 30 | -54 | 60 | MNI | Nee et al. (2013), Supplemental Table II |
|  |  | 32 | -52 | 46 | MNI | Nee et al. (2013), Supplemental Table II |
|  |  | 18 | -62 | 48 | MNI | Nee et al. (2007) |
|  |  | 6 | -68 | 48 | MNI | Nee et al. (2007) |
|  |  | 10 | -66 | 48 | TAL | Owen et al. (2005) |
|  |  | 31 | -59 | 43 | MNI | Wager and Smith (2003) |
| IPL | L | -42 | -46 | 46 | TAL | Kim et al. (2012) |
|  |  | -44 | -38 | 42 | TAL | Kim et al. (2012) |
|  |  | -40 | -54 | 52 | MNI | Nee et al. (2013), Supplemental Table I |
|  |  | -58 | -38 | 50 | MNI | Nee et al. (2013), Supplemental Table I |
|  |  | -38 | -44 | 48 | MNI | Nee et al. (2013), Supplemental Table II |
|  |  | -40 | -52 | 52 | MNI | Nee et al. (2013), Supplemental Table II |
|  |  | -56 | -38 | 48 | MNI | Nee et al. (2013), Supplemental Table II |
|  |  | -58 | -38 | 32 | MNI | Nee et al. (2013), Supplemental Table II |
|  |  | -36 | -56 | 44 | MNI | Nee et al. (2007) |
|  |  | -36 | -50 | 40 | TAL | Owen et al. (2005) |
|  |  | -37 | -51 | 41 | MNI | Wager and Smith (2003) |
| IPL | R | 38 | -44 | 43 | TAL | Derfuss et al. (2004) |
|  |  | 36 | -46 | 40 | TAL | Kim et al. (2012) |
|  |  | 36 | -54 | 46 | MNI | Nee et al. (2013), Supplemental Table II |
|  |  | 42 | -40 | 50 | MNI | Nee et al. (2013), Supplemental Table II |
|  |  | 40 | -52 | 42 | MNI | Nee et al. (2007) |
|  |  | 46 | -48 | 38 | MNI | Nee et al. (2007) |
|  |  | 50 | -44 | 32 | MNI | Nee et al. (2007) |
|  |  | 36 | -54 | 48 | MNI | Nee et al. (2007) |
|  |  | 40 | -48 | 38 | TAL | Owen et al. (2005) |
|  |  | 58 | -36 | 44 | TAL | Owen et al. (2005) |

ROI = Region of interest; BA = Brodmann area; Hemi = hemisphere; FP = frontal pole; ACC = anterior cingulate cortex; BG = basal ganglia; DLPFC = dorsolateral prefrontal cortex; Mid-VLPFC = ventrolateral prefrontal cortex; IFJ = inferior frontal junction; SPL = superior parietal lobule; IPL = inferior parietal lobule; L = left; R = right.

**References for supplementary material (meta-analyses)**

Ashburner, J., & Friston, K. J. (2011). Diffeomorphic registration using geodesic shooting and Gauss–Newton optimisation. *NeuroImage, 55*(3), 954-967. doi:10.1016/j.neuroimage.2010.12.049

Bartra, O., McGuire, J. T., & Kable, J. W. (2013). The valuation system: a coordinate-based meta-analysis of BOLD fMRI experiments examining neural correlates of subjective value. *NeuroImage, 76*, 412-427. doi:10.1016/j.neuroimage.2013.02.063

Derrfuss, J., Brass, M., Neumann, J., & von Cramon, D. Y. (2005). Involvement of the inferior frontal junction in cognitive control: Meta‐analyses of switching and Stroop studies. *Human brain mapping, 25*(1), 22-34. doi:10.1002/hbm.20127

Derrfuss, J., Brass, M., & Von Cramon, D. Y. (2004). Cognitive control in the posterior frontolateral cortex: evidence from common activations in task coordination, interference control, and working memory. *NeuroImage, 23*(2), 604-612.

Diekhof, E. K., Kaps, L., Falkai, P., & Gruber, O. (2012). The role of the human ventral striatum and the medial orbitofrontal cortex in the representation of reward magnitude-an activation likelihood estimation meta-analysis of neuroimaging studies of passive reward expectancy and outcome processing. *Neuropsychologia, 50*(7), 1252-1266.

Eickhoff, S. B., Stephan, K., Mohlberg, H., Grefkes, C., Fink, G. R., Amunts, K., & Zilles, K. (2005). A new SPM toolbox for combining probabilistic cytoarchitectonic maps and functional imaging data. *NeuroImage, 25*(4), 1325-1335.

Garrison, J., Erdeniz, B., & Done, J. (2013). Prediction error in reinforcement learning: a meta-analysis of neuroimaging studies. *Neuroscience & Biobehavioral Reviews, 37*(7), 1297-1310.

Gilbert, S. J., Spengler, S., Simons, J. S., Steele, J. D., Lawrie, S. M., Frith, C. D., & Burgess, P. W. (2006). Functional specialization within rostral prefrontal cortex (area 10): a meta-analysis. *Journal of cognitive neuroscience, 18*(6), 932-948.

Groenewold, N. A., Opmeer, E. M., de Jonge, P., Aleman, A., & Costafreda, S. G. (2013). Emotional valence modulates brain functional abnormalities in depression: evidence from a meta-analysis of fMRI studies. *Neuroscience & Biobehavioral Reviews, 37*(2), 152-163. doi:10.1016/j.neubiorev.2012.11.015

Jamadar, S., Fielding, J., & Egan, G. (2013). Quantitative meta-analysis of fMRI and PET studies reveals consistent activation in fronto-striatal-parietal regions and cerebellum during antisaccades and prosaccades. *Frontiers in psychology, 4*, 749.

Kaps, L. (2012). *Verarbeitung des relativen Belohnungswertes im menschlichen Gehirn. Eine Metaanalyse hirnbildgebender Studien.* (Unpublished doctoral dissertation), Georg-August-Universität, Göttingen. Retrieved from <http://hdl.handle.net/11858/00-1735-0000-0006-B2CF-8>

Kim, C., Cilles, S. E., Johnson, N. F., & Gold, B. T. (2012). Domain general and domain preferential brain regions associated with different types of task switching: a meta-analysis. *Human brain mapping, 33*(1), 130-142.

Liu, X., Hairston, J., Schrier, M., & Fan, J. (2011). Common and distinct networks underlying reward valence and processing stages: a meta-analysis of functional neuroimaging studies. *Neuroscience & Biobehavioral Reviews, 35*(5), 1219-1236. doi:10.1016/j.neubiorev.2010.12.012

Murty, V. P., Ritchey, M., Adcock, R. A., & LaBar, K. S. (2010). fMRI studies of successful emotional memory encoding: A quantitative meta-analysis. *Neuropsychologia, 48*(12), 3459-3469. doi:10.1016/j.neuropsychologia.2010.07.030

Nee, D. E., Brown, J. W., Askren, M. K., Berman, M. G., Demiralp, E., Krawitz, A., & Jonides, J. (2013). A meta-analysis of executive components of working memory. *Cerebral Cortex, 23*(2), 264-282. doi:10.1093/cercor/bhs007

Nee, D. E., Wager, T. D., & Jonides, J. (2007). Interference resolution: insights from a meta-analysis of neuroimaging tasks. *Cognitive, Affective, & Behavioral Neuroscience, 7*(1), 1-17.

Owen, A. M., McMillan, K. M., Laird, A. R., & Bullmore, E. (2005). N‐back working memory paradigm: A meta‐analysis of normative functional neuroimaging studies. *Human brain mapping, 25*(1), 46-59. doi:10.1002/hbm.20131

Schubert, R., Ritter, P., Wüstenberg, T., Preuschhof, C., Curio, G., Sommer, W., & Villringer, A. (2008). Spatial attention related SEP amplitude modulations covary with BOLD signal in S1—a simultaneous EEG—fMRI study. *Cerebral Cortex, 18*(11), 2686-2700. doi:10.1093/cercor/bhn029

Wager, T. D., Jonides, J., & Reading, S. (2004). Neuroimaging studies of shifting attention: a meta-analysis. *NeuroImage, 22*(4), 1679-1693.

Wager, T. D., & Smith, E. E. (2003). Neuroimaging studies of working memory. *Cognitive, Affective, & Behavioral Neuroscience, 3*(4), 255-274.

**SI3.** Region-wise results for the group x session x condition crossover interaction in the context-updating task.

|  | ROI | Included BA | Hemi | Interaction effect *group x session x condition* | | |  |
| --- | --- | --- | --- | --- | --- | --- | --- |
|  |  |  |  | *F* | *df* | *η*_p_² | *p* |
| *Transient-sensitive task-switching inclusion mask* | Mid-VlPFC | 44,45 | L | 0.07 | 42 | .00 | .79 |
|  |  |  | R | 1.10 | 42 | .03 | .30 |
|  | IFJ | 6,8,9,44 (intersection) | L | 0.04 | 42 | .00 | .85 |
|  |  |  | R | 4.42 | 42 | .10 | .04 |
|  | SPL | 7 | L | 0.22 | 42 | .01 | .32 |
| *Sustained-sensitive task-switching inclusion mask* | BG (putamen, caudate nucleus) | n. a. | L | 0.15 | 42 | .00 | .35 |
|  |  |  | R | 0.72 | 42 | .02 | .40 |
| *Task-switching exclusion mask* | FP | 10,11 | L | 1.90 | 42 | .04 | .09 |
|  |  |  | R | 0.30 | 42 | .01 | .29 |
|  | ACC | 6,8,32 | L | 2.14 | 42 | .05 | .15 |
|  |  |  | R | 2.16 | 42 | .05 | .15 |
|  | DlPFC | 9,46 | L | 2.74 | 42 | .06 | .11 |
|  |  |  | R | 3.44 | 42 | .08 | .07 |
|  | SPL |  | R | 0.96 | 42 | .02 | .33 |
|  | IPL | 40 | L | 4.01 | 42 | .09 | .03 |
|  |  |  | R | 2.83 | 42 | .06 | .10 |

ROI = Region of interest; BA = Brodmann area; Hemi = hemisphere; FP = frontal pole; ACC = anterior cingulate cortex; BG = basal ganglia; DLPFC = dorsolateral prefrontal cortex; Mid-VLPFC = ventrolateral prefrontal cortex; IFJ = inferior frontal junction; SPL = superior parietal lobule; IPL = inferior parietal lobule; L = left; R = right.

**SI4.** Region-wise results for the group x session x condition crossover interaction in the delayed-recognition task.

|  | ROI | Included BA | Hemi | Interaction effect *group x session x condition* | | |  |
| --- | --- | --- | --- | --- | --- | --- | --- |
|  |  |  |  | *F* | *df* | *η*_p_² | *p* |
| *Transient-sensitive task-switching inclusion mask* | Mid-VlPFC | 44,45 | L | 0.52 | 80 | .01 | .57 |
|  |  |  | R | 0.86 | 80 | .02 | .40 |
|  | IFJ | 6,8,9,44 (intersection) | L | 1.11 | 80 | .03 | .33 |
|  |  |  | R | 2.18 | 80 | .05 | .12 |
|  | SPL | 7 | L | 2.32 | 80 | .06 | .05 |
| *Sustained-sensitive task-switching inclusion mask* | BG (putamen, caudate nucleus) | n. a. | L | 4.68 | 80 | .11 | .01 |
|  |  |  | R | 2.78 | 80 | .07 | .04 |
| *Task-switching exclusion mask* | FP | 10,11 | L | 1.17 | 80 | .03 | .31 |
|  |  |  | R | 1.34 | 80 | .03 | .13 |
|  | ACC | 6,8,32 | L | 0.64 | 80 | .02 | .25 |
|  |  |  | R | 0.36 | 80 | .01 | .65 |
|  | DlPFC | 9,46 | L | 0.47 | 80 | .01 | .62 |
|  |  |  | R | 1.18 | 80 | .03 | .31 |
|  | SPL |  | R | 1.27 | 80 | .03 | .29 |
|  | IPL | 40 | L | 0.91 | 80 | .02 | .20 |
|  |  |  | R | 2.13 | 80 | .05 | .06 |

ROI = Region of interest; BA = Brodmann area; Hemi = hemisphere; FP = frontal pole; ACC = anterior cingulate cortex; BG = basal ganglia; DLPFC = dorsolateral prefrontal cortex; Mid-VLPFC = ventrolateral prefrontal cortex; IFJ = inferior frontal junction; SPL = superior parietal lobule; IPL = inferior parietal lobule; L = left; R = right.

**SI5.** Region-wise BOLD signal change values (%) as a function of training group (single-task active control group/ CG, task-switching training group/ TG), task (context-updating task, delayed-recognition task), hemisphere (left, right), session (pretest, posttest), and condition (context-updating task: context-independent/ c-indep, context-dependent/ c-dep; delayed-recognition task: passive viewing/ PV, distraction/ DS, interruption/ IS).

| **Mid-VlPFC** |  |  | Group | | | | |
| --- | --- | --- | --- | --- | --- | --- | --- |
|  |  |  | Single-task active control group (CG) | | Task-switching training group  (TG) | | |
| *Context-updating task* |  |  | left hemisphere | | | | |
|  |  |  | *M* | *SD* | | *M* | *SD* |
|  | Pretest | c-indep | 0.03 | 0.23 | | 0.08 | 0.16 |
|  |  | c-dep | 0.07 | 0.19 | | 0.07 | 0.19 |
|  | Posttest | c-indep | -0.02 | 0.17 | | -0.04 | 0.15 |
|  |  | c-dep | 0.08 | 0.23 | | 0.00 | 0.19 |
|  |  |  | right hemisphere | | | | |
|  |  |  | *M* | *SD* | | *M* | *SD* |
|  | Pretest | c-indep | 0.04 | 0.14 | | 0.04 | 0.11 |
|  |  | c-dep | 0.07 | 0.12 | | 0.05 | 0.17 |
|  | Posttest | c-indep | -0.02 | 0.19 | | -0.02 | 0.12 |
|  |  | c-dep | 0.08 | 0.16 | | 0.00 | 0.16 |
|  |  |  |  |  | |  |  |
| *Delayed-recognition task* |  |  | left hemisphere | | | | |
|  |  |  | *M* | *SD* | | *M* | *SD* |
|  | Pretest | PV | -0.07 | 0.61 | | -0.26 | 0.92 |
|  |  | DS | -0.02 | 1.36 | | 0.16 | 1.27 |
|  |  | IS | 0.00 | 1.02 | | 0.33 | 0.73 |
|  | Posttest | PV | 0.12 | 0.63 | | 0.19 | 1.06 |
|  |  | DS | 0.00 | 0.67 | | 0.26 | 0.75 |
|  |  | IS | 0.34 | 0.74 | | 0.45 | 0.97 |
|  |  |  | right hemisphere | | | | |
|  |  |  | *M* | *SD* | | *M* | *SD* |
|  | Pretest | PV | 0.17 | 0.69 | | 0.02 | 0.39 |
|  |  | DS | 0.11 | 0.58 | | 0.09 | 0.67 |
|  |  | IS | 0.06 | 0.67 | | 0.23 | 0.66 |
|  | Posttest | PV | 0.31 | 0.64 | | -0.12 | 1.35 |
|  |  | DS | 0.00 | 0.56 | | 0.28 | 0.82 |
|  |  | IS | 0.20 | 0.49 | | 0.25 | 0.71 |

M = mean; SD = standard deviation. Mid-VlPFC = mid-ventrolateral prefrontal cortex.

| **IFJ** |  |  | Group | | | | |
| --- | --- | --- | --- | --- | --- | --- | --- |
|  |  |  | Single-task active control group (CG) | | Task-switching training group  (TG) | | |
| *Context-updating task* |  |  | left hemisphere | | | | |
|  |  |  | *M* | *SD* | | *M* | *SD* |
|  | Pretest | c-indep | 0.00 | 0.20 | | 0.05 | 0.10 |
|  |  | c-dep | 0.03 | 0.14 | | 0.08 | 0.13 |
|  | Posttest | c-indep | 0.00 | 0.18 | | -0.06 | 0.17 |
|  |  | c-dep | 0.05 | 0.22 | | -0.02 | 0.17 |
|  |  |  | right hemisphere | | | | |
|  |  |  | *M* | *SD* | | *M* | *SD* |
|  | Pretest | c-indep | 0.02 | 0.22 | | 0.02 | 0.10 |
|  |  | c-dep | 0.01 | 0.10 | | 0.05 | 0.10 |
|  | Posttest | c-indep | -0.04 | 0.18 | | 0.02 | 0.18 |
|  |  | c-dep | 0.08 | 0.22 | | 0.04 | 0.19 |
|  |  |  |  |  | |  |  |
| *Delayed-recognition task* |  |  | left hemisphere | | | | |
|  |  |  | *M* | *SD* | | *M* | *SD* |
|  | Pretest | PV | 0.01 | 0.46 | | -0.08 | 0.54 |
|  |  | DS | 0.25 | 0.62 | | 0.27 | 0.55 |
|  |  | IS | 0.28 | 0.80 | | 0.51 | 0.80 |
|  | Posttest | PV | 0.29 | 0.57 | | 0.06 | 0.92 |
|  |  | DS | 0.21 | 0.65 | | 0.56 | 0.60 |
|  |  | IS | 0.53 | 0.97 | | 0.62 | 0.75 |
|  |  |  | right hemisphere | | | | |
|  |  |  | *M* | *SD* | | *M* | *SD* |
|  | Pretest | PV | 0.08 | 0.70 | | 0.22 | 0.79 |
|  |  | DS | 0.30 | 0.75 | | 0.18 | 0.84 |
|  |  | IS | 0.01 | 0.57 | | 0.27 | 0.65 |
|  | Posttest | PV | 0.21 | 0.59 | | -0.05 | 0.82 |
|  |  | DS | 0.09 | 0.45 | | 0.26 | 0.58 |
|  |  | IS | 0.25 | 0.75 | | 0.25 | 0.85 |

M = mean; SD = standard deviation. IFJ = inferior frontal junction.

| **SPL** |  |  | Group | | | | |
| --- | --- | --- | --- | --- | --- | --- | --- |
|  |  |  | Single-task active control group (CG) | | Task-switching training group  (TG) | | |
| *Context-updating task* |  |  | left hemisphere | | | | |
|  |  |  | *M* | *SD* | | *M* | *SD* |
|  | Pretest | c-indep | 0.05 | 0.25 | | 0.10 | 0.18 |
|  |  | c-dep | 0.12 | 0.23 | | 0.11 | 0.19 |
|  | Posttest | c-indep | 0.04 | 0.20 | | 0.07 | 0.15 |
|  |  | c-dep | 0.10 | 0.22 | | 0.11 | 0.18 |
|  |  |  | right hemisphere | | | | |
|  |  |  | *M* | *SD* | | *M* | *SD* |
|  | Pretest | c-indep | 0.08 | 0.31 | | 0.09 | 0.15 |
|  |  | c-dep | 0.08 | 0.22 | | 0.10 | 0.19 |
|  | Posttest | c-indep | -0.01 | 0.36 | | 0.04 | 0.15 |
|  |  | c-dep | 0.16 | 0.31 | | 0.09 | 0.21 |
|  |  |  |  |  | |  |  |
| *Delayed-recognition task* |  |  | left hemisphere | | | | |
|  |  |  | *M* | *SD* | | *M* | *SD* |
|  | Pretest | PV | 0.02 | 0.56 | | 0.13 | 1.40 |
|  |  | DS | 0.37 | 0.60 | | -0.16 | 0.92 |
|  |  | IS | 0.19 | 0.86 | | 0.32 | 0.72 |
|  | Posttest | PV | 0.17 | 0.60 | | 0.05 | 0.66 |
|  |  | DS | 0.11 | 0.56 | | 0.39 | 0.83 |
|  |  | IS | 0.10 | 0.87 | | 0.43 | 1.16 |
|  |  |  | right hemisphere | | | | |
|  |  |  | *M* | *SD* | | *M* | *SD* |
|  | Pretest | PV | 0.12 | 0.78 | | -0.21 | 0.71 |
|  |  | DS | 0.28 | 0.88 | | 0.09 | 0.61 |
|  |  | IS | 0.19 | 0.83 | | 0.32 | 0.72 |
|  | Posttest | PV | 0.19 | 0.81 | | 0.26 | 0.85 |
|  |  | DS | 0.13 | 0.58 | | 0.52 | 0.96 |
|  |  | IS | 0.19 | 0.68 | | 0.28 | 1.06 |

M = mean; SD = standard deviation. SPL = superior parietal lobule.

| **BG** |  |  | Group | | | | |
| --- | --- | --- | --- | --- | --- | --- | --- |
|  |  |  | Single-task active control group (CG) | | Task-switching training group  (TG) | | |
| *Context-updating task* |  |  | left hemisphere | | | | |
|  |  |  | *M* | *SD* | | *M* | *SD* |
|  | Pretest | c-indep | 0.06 | 0.12 | | 0.07 | 0.11 |
|  |  | c-dep | 0.05 | 0.10 | | 0.03 | 0.12 |
|  | Posttest | c-indep | 0.08 | 0.16 | | 0.05 | 0.09 |
|  |  | c-dep | 0.11 | 0.16 | | 0.05 | 0.12 |
|  |  |  | right hemisphere | | | | |
|  |  |  | *M* | *SD* | | *M* | *SD* |
|  | Pretest | c-indep | 0.07 | 0.18 | | 0.08 | 0.12 |
|  |  | c-dep | 0.03 | 0.14 | | 0.05 | 0.19 |
|  | Posttest | c-indep | 0.04 | 0.25 | | 0.07 | 0.11 |
|  |  | c-dep | 0.11 | 0.20 | | 0.07 | 0.16 |
|  |  |  |  |  | |  |  |
| *Delayed-recognition task* |  |  | left hemisphere | | | | |
|  |  |  | *M* | *SD* | | *M* | *SD* |
|  | Pretest | PV | 0.09 | 0.53 | | 0.10 | 0.70 |
|  |  | DS | 0.39 | 0.93 | | -0.01 | 0.60 |
|  |  | IS | 0.03 | 0.66 | | 0.16 | 0.46 |
|  | Posttest | PV | -0.03 | 0.58 | | -0.14 | 0.74 |
|  |  | DS | -0.15 | 0.45 | | 0.21 | 0.63 |
|  |  | IS | 0.09 | 0.42 | | 0.07 | 0.60 |
|  |  |  | right hemisphere | | | | |
|  |  |  | *M* | *SD* | | *M* | *SD* |
|  | Pretest | PV | 0.03 | 0.35 | | 0 | 0.36 |
|  |  | DS | 0.27 | 0.57 | | -0.15 | 0.5 |
|  |  | IS | 0.13 | 0.44 | | 0.17 | 0.5 |
|  | Posttest | PV | -0.13 | 0.86 | | -0.45 | 1.4 |
|  |  | DS | -0.08 | 0.43 | | 0.19 | 0.69 |
|  |  | IS | 0.02 | 0.33 | | 0.14 | 0.39 |

M = mean; SD = standard deviation. BG = basal ganglia.

| **FP** |  |  | Group | | | | |
| --- | --- | --- | --- | --- | --- | --- | --- |
|  |  |  | Single-task active control group (CG) | | Task-switching training group  (TG) | | |
| *Context-updating task* |  |  | left hemisphere | | | | |
|  |  |  | *M* | *SD* | | *M* | *SD* |
|  | Pretest | c-indep | 0.06 | 0.31 | | 0.10 | 0.13 |
|  |  | c-dep | -0.02 | 0.17 | | 0.10 | 0.17 |
|  | Posttest | c-indep | -0.04 | 0.17 | | 0.02 | 0.15 |
|  |  | c-dep | 0.06 | 0.20 | | 0.08 | 0.23 |
|  |  |  | right hemisphere | | | | |
|  |  |  | *M* | *SD* | | *M* | *SD* |
|  | Pretest | c-indep | 0.04 | 0.17 | | 0.05 | 0.11 |
|  |  | c-dep | 0.01 | 0.13 | | 0.05 | 0.12 |
|  | Posttest | c-indep | -0.01 | 0.09 | | -0.01 | 0.12 |
|  |  | c-dep | 0.03 | 0.16 | | 0.03 | 0.15 |
|  |  |  |  |  | |  |  |
| *Delayed-recognition task* |  |  | left hemisphere | | | | |
|  |  |  | *M* | *SD* | | *M* | *SD* |
|  | Pretest | PV | 0.03 | 0.65 | | 0.10 | 1.03 |
|  |  | DS | 0.24 | 1.26 | | 0.11 | 0.67 |
|  |  | IS | 0.09 | 0.72 | | 0.10 | 0.55 |
|  | Posttest | PV | -0.04 | 0.50 | | -0.13 | 0.38 |
|  |  | DS | -0.22 | 0.68 | | 0.07 | 0.48 |
|  |  | IS | 0.04 | 0.52 | | 0.13 | 0.48 |
|  |  |  | right hemisphere | | | | |
|  |  |  | *M* | *SD* | | *M* | *SD* |
|  | Pretest | PV | 0.06 | 0.62 | | 0.06 | 0.59 |
|  |  | DS | 0.15 | 0.64 | | -0.03 | 0.39 |
|  |  | IS | 0.02 | 0.55 | | 0.10 | 0.52 |
|  | Posttest | PV | -0.12 | 0.49 | | -0.30 | 0.99 |
|  |  | DS | -0.11 | 0.56 | | 0.12 | 0.53 |
|  |  | IS | -0.03 | 0.49 | | 0.14 | 0.38 |

M = mean; SD = standard deviation. FP = frontal pole.

| **ACC** |  |  | Group | | | | |
| --- | --- | --- | --- | --- | --- | --- | --- |
|  |  |  | Single-task active control group (CG) | | Task-switching training group  (TG) | | |
| *Context-updating task* |  |  | left hemisphere | | | | |
|  |  |  | *M* | *SD* | | *M* | *SD* |
|  | Pretest | c-indep | 0.02 | 0.17 | | 0.06 | 0.09 |
|  |  | c-dep | 0.04 | 0.11 | | 0.06 | 0.12 |
|  | Posttest | c-indep | -0.05 | 0.19 | | 0.02 | 0.14 |
|  |  | c-dep | 0.06 | 0.19 | | 0.02 | 0.14 |
|  |  |  | right hemisphere | | | | |
|  |  |  | *M* | *SD* | | *M* | *SD* |
|  | Pretest | c-indep | 0.04 | 0.18 | | 0.05 | 0.10 |
|  |  | c-dep | 0.07 | 0.11 | | 0.07 | 0.12 |
|  | Posttest | c-indep | -0.05 | 0.21 | | 0.04 | 0.17 |
|  |  | c-dep | 0.05 | 0.16 | | 0.04 | 0.15 |
|  |  |  |  |  | |  |  |
| *Delayed-recognition task* |  |  | left hemisphere | | | | |
|  |  |  | *M* | *SD* | | *M* | *SD* |
|  | Pretest | PV | 0.16 | 0.46 | | 0.12 | 0.35 |
|  |  | DS | 0.40 | 0.84 | | 0.33 | 0.56 |
|  |  | IS | 0.29 | 0.45 | | 0.43 | 0.65 |
|  | Posttest | PV | 0.00 | 0.57 | | 0.03 | 1.01 |
|  |  | DS | 0.11 | 0.42 | | 0.46 | 0.43 |
|  |  | IS | 0.23 | 0.59 | | 0.43 | 0.53 |
|  |  |  | right hemisphere | | | | |
|  |  |  | *M* | *SD* | | *M* | *SD* |
|  | Pretest | PV | 0.12 | 0.44 | | 0.15 | 0.39 |
|  |  | DS | 0.33 | 0.83 | | 0.39 | 0.68 |
|  |  | IS | 0.32 | 0.42 | | 0.42 | 0.57 |
|  | Posttest | PV | 0.07 | 0.54 | | 0.06 | 1.02 |
|  |  | DS | 0.14 | 0.44 | | 0.47 | 0.48 |
|  |  | IS | 0.29 | 0.55 | | 0.52 | 0.77 |

M = mean; SD = standard deviation. ACC = anterior cingulate cortex.

| **DLPFC** |  |  | Group | | | | |
| --- | --- | --- | --- | --- | --- | --- | --- |
|  |  |  | Single-task active control group (CG) | | Task-switching training group  (TG) | | |
| *Context-updating task* |  |  | left hemisphere | | | | |
|  |  |  | *M* | *SD* | | *M* | *SD* |
|  | Pretest | c-indep | 0.07 | 0.16 | | 0.08 | 0.09 |
|  |  | c-dep | 0.06 | 0.10 | | 0.11 | 0.10 |
|  | Posttest | c-indep | 0.00 | 0.13 | | 0.00 | 0.10 |
|  |  | c-dep | 0.08 | 0.15 | | 0.05 | 0.11 |
|  |  |  | right hemisphere | | | | |
|  |  |  | *M* | *SD* | | *M* | *SD* |
|  | Pretest | c-indep | 0.03 | 0.22 | | 0.08 | 0.12 |
|  |  | c-dep | 0.04 | 0.12 | | 0.12 | 0.11 |
|  | Posttest | c-indep | -0.02 | 0.20 | | 0.05 | 0.23 |
|  |  | c-dep | 0.11 | 0.19 | | 0.08 | 0.24 |
|  |  |  |  |  | |  |  |
| *Delayed-recognition task* |  |  | left hemisphere | | | | |
|  |  |  | *M* | *SD* | | *M* | *SD* |
|  | Pretest | PV | 0.09 | 0.50 | | -0.04 | 0.41 |
|  |  | DS | 0.25 | 0.62 | | 0.16 | 0.67 |
|  |  | IS | 0.19 | 0.47 | | 0.20 | 0.45 |
|  | Posttest | PV | 0.02 | 0.46 | | 0.05 | 0.61 |
|  |  | DS | 0.02 | 0.51 | | 0.23 | 0.43 |
|  |  | IS | 0.30 | 0.85 | | 0.31 | 0.50 |
|  |  |  | right hemisphere | | | | |
|  |  |  | *M* | *SD* | | *M* | *SD* |
|  | Pretest | PV | 0.06 | 0.76 | | 0.24 | 0.93 |
|  |  | DS | 0.17 | 0.81 | | 0.32 | 0.95 |
|  |  | IS | -0.03 | 0.64 | | 0.47 | 0.55 |
|  | Posttest | PV | 0.31 | 0.58 | | 0.19 | 0.70 |
|  |  | DS | -0.05 | 0.70 | | 0.30 | 0.58 |
|  |  | IS | 0.28 | 0.94 | | 0.42 | 0.58 |

M = mean; SD = standard deviation. DLPFC = dorsolateral prefrontal cortex.

| **IPL** |  |  | Group | | | |
| --- | --- | --- | --- | --- | --- | --- |
|  |  |  | Single-task active control group (CG) | | Task-switching training group (TG) | |
| *Context-updating task* |  |  | left hemisphere | | | |
|  |  |  | *M* | *SD* | *M* | *SD* |
|  | Pretest | c-indep | 0.02 | 0.23 | 0.10 | 0.13 |
|  |  | c-dep | 0.09 | 0.18 | 0.03 | 0.18 |
|  | Posttest | c-indep | 0.06 | 0.13 | 0.02 | 0.18 |
|  |  | c-dep | 0.03 | 0.18 | 0.01 | 0.20 |
|  |  |  | right hemisphere | | | |
|  |  |  | *M* | *SD* | *M* | *SD* |
|  | Pretest | c-indep | 0.06 | 0.21 | 0.06 | 0.13 |
|  |  | c-dep | 0.06 | 0.13 | 0.11 | 0.14 |
|  | Posttest | c-indep | 0.00 | 0.32 | 0.07 | 0.22 |
|  |  | c-dep | 0.17 | 0.31 | 0.07 | 0.22 |
|  |  |  |  |  |  |  |
| *Delayed-recognition task* |  |  | left hemisphere | | | |
|  |  |  | *M* | *SD* | *M* | *SD* |
|  | Pretest | PV | 0.02 | 0.47 | -0.07 | 0.70 |
|  |  | DS | -0.19 | 1.21 | 0.00 | 0.84 |
|  |  | IS | 0.03 | 0.53 | 0.36 | 0.74 |
|  | Posttest | PV | 0.12 | 0.60 | 0.11 | 0.62 |
|  |  | DS | 0.23 | 0.58 | 0.17 | 0.55 |
|  |  | IS | 0.34 | 0.58 | 0.28 | 0.39 |
|  |  |  | right hemisphere | | | |
|  |  |  | *M* | *SD* | *M* | *SD* |
|  | Pretest | PV | 0.14 | 0.73 | 0.18 | 0.96 |
|  |  | DS | 0.16 | 0.61 | 0.10 | 0.91 |
|  |  | IS | 0.05 | 0.65 | 0.23 | 0.63 |
|  | Posttest | PV | 0.15 | 0.53 | 0.16 | 0.71 |
|  |  | DS | 0.00 | 0.61 | 0.39 | 0.60 |
|  |  | IS | 0.16 | 0.60 | 0.14 | 0.63 |

M = mean; SD = standard deviation. IPL = inferior parietal lobule.

**SI6.** Region-wise values for the absolute magnitude of context-updating costs activation in the context-updating task.

|  |  | Group | | | | | |
| --- | --- | --- | --- | --- | --- | --- | --- |
| *ROI* |  | *Single-task active control group (CG)* | | | *Task-switching training group (TG)* | | |
|  |  | Absolute magnitude of context-updating costs activation | | | | | |
|  |  | *Pretest* | *Posttest* | | *Pretest* | | *Posttest* |
| Mid-VlPFC | L | 0.04 | | 0.10 | 0.01 | 0.04 | |
|  | R | 0.03 | | 0.1 | 0.01 | 0.02 | |
| IFJ | L | 0.03 | | 0.05 | 0.03 | 0.04 | |
|  | R | 0.01 | | 0.12 | 0.03 | 0.02 | |
| SPL | L | 0.07 | | 0.06 | 0.01 | 0.04 | |
|  | R | 0.00 | | 0.17 | 0.01 | 0.05 | |
| BG | L | 0.01 | | 0.03 | 0.04 | 0.00 | |
|  | R | 0.04 | | 0.07 | 0.03 | 0.00 | |
| FP | L | 0.08 | | 0.10 | 0.00 | 0.06 | |
|  | R | 0.03 | | 0.04 | 0.00 | 0.04 | |
| ACC | L | 0.02 | | 0.11 | 0.00 | 0.00 | |
|  | R | 0.03 | | 0.10 | 0.02 | 0.00 | |
| DlPFC | L | 0.01 | | 0.08 | 0.03 | 0.05 | |
|  | R | 0.01 | | 0.13 | 0.04 | 0.03 | |
| IPL | L | 0.07 | | 0.03 | 0.07 | 0.01 | |
|  | R | 0.00 | | 0.17 | 0.05 | 0.00 | |

ROI = Region of interest; BA = Brodmann area; Hemi = hemisphere; FP = frontal pole; ACC = anterior cingulate cortex; BG = basal ganglia; DLPFC = dorsolateral prefrontal cortex; Mid-VLPFC = ventrolateral prefrontal cortex; IFJ = inferior frontal junction; SPL = superior parietal lobule; IPL = inferior parietal lobule; L = left; R = right.

**SI7.** Region-wise values for the absolute magnitude of WM maintenance and scheduling costs activation in the delayed-recognition task.

|  |  | Group | | | |  |  |  |  |
| --- | --- | --- | --- | --- | --- | --- | --- | --- | --- |
| *ROI* |  | *Single-task active control group (CG)* | | *Task-switching training group (TG)* | | *Single-task active control group (CG)* | | *Task-switching training group (TG)* | |
|  |  | Absolute magnitude of WM maintenance costs activation | | | | Absolute magnitude of WM scheduling costs activation | | | |
|  |  | *Pretest* | *Posttest* | *Pretest* | *Posttest* | *Pretest* | *Posttest* | *Pretest* | *Posttest* |
| Mid-VlPFC | L | 0.06 | 0.05 | 0.51 | 0.17 | 0.02 | 0.34 | 0.17 | 0.19 |
|  | R | 0.09 | 0.21 | 0.14 | 0.39 | 0.05 | 0.20 | 0.14 | 0.03 |
| IFJ | L | 0.26 | 0.08 | 0.47 | 0.53 | 0.03 | 0.32 | 0.24 | 0.06 |
|  | R | 0.08 | 0.04 | 0.01 | 0.31 | 0.29 | 0.16 | 0.09 | 0.01 |
| SPL | L | 0.26 | 0.07 | 0.05 | 0.36 | 0.18 | 0.01 | 0.48 | 0.04 |
|  | R | 0.12 | 0.03 | 0.42 | 0.14 | 0.09 | 0.06 | 0.23 | 0.24 |
| BG | L | 0.12 | 0.00 | 0.03 | 0.28 | 0.36 | 0.24 | 0.17 | 0.14 |
|  | R | 0.17 | 0.10 | 0.02 | 0.61 | 0.14 | 0.09 | 0.32 | 0.05 |
| FP | L | 0.14 | 0.05 | 0.01 | 0.23 | 0.15 | 0.26 | 0.01 | 0.06 |
|  | R | 0.03 | 0.05 | 0.03 | 0.43 | 0.13 | 0.08 | 0.13 | 0.02 |
| ACC | L | 0.19 | 0.17 | 0.26 | 0.42 | 0.11 | 0.12 | 0.10 | 0.03 |
|  | R | 0.21 | 0.15 | 0.26 | 0.44 | 0.01 | 0.15 | 0.03 | 0.05 |
| DlPFC | L | 0.13 | 0.14 | 0.22 | 0.22 | 0.06 | 0.28 | 0.04 | 0.08 |
|  | R | 0.01 | 0.20 | 0.16 | 0.17 | 0.20 | 0.33 | 0.15 | 0.12 |
| IPL | L | 0.10 | 0.17 | 0.25 | 0.12 | 0.22 | 0.11 | 0.36 | 0.11 |
|  | R | 0.04 | 0.07 | 0.02 | 0.11 | 0.11 | 0.16 | 0.13 | 0.25 |

ROI = Region of interest; BA = Brodmann area; Hemi = hemisphere; FP = frontal pole; ACC = anterior cingulate cortex; BG = basal ganglia; DLPFC = dorsolateral prefrontal cortex; Mid-VLPFC = ventrolateral prefrontal cortex; IFJ = inferior frontal junction; SPL = superior parietal lobule; IPL = inferior parietal lobule; L = left; R = right.
